# Segmenting surface boundaries using luminance cues

**DOI:** 10.1038/s41598-021-89277-2

**Published:** 2021-05-12

**Authors:** Christopher DiMattina, Curtis L. Baker

**Affiliations:** 1 Computational Perception Laboratory & Department of Psychology, Florida Gulf Coast University, Whitaker Hall Room 215, 10501 FGCU Blvd S., Fort Myers, FL 33965-6565 USA; 2 McGill Vision Research Unit, Department of Ophthalmology and Visual Sciences, McGill University, Montreal, QC H3G1A4 Canada

**Keywords:** Neuroscience, Psychology

## Abstract

Segmenting scenes into distinct surfaces is a basic visual perception task, and luminance differences between adjacent surfaces often provide an important segmentation cue. However, mean luminance differences between two surfaces may exist without any sharp change in albedo at their boundary, but rather from differences in the proportion of small light and dark areas within each surface, e.g. texture elements, which we refer to as a *luminance texture boundary*. Here we investigate the performance of human observers segmenting luminance texture boundaries. We demonstrate that a simple model involving a single stage of filtering cannot explain observer performance, unless it incorporates contrast normalization. Performing additional experiments in which observers segment luminance texture boundaries while ignoring super-imposed luminance step boundaries, we demonstrate that the one-stage model, even with contrast normalization, cannot explain performance. We then present a Filter–Rectify–Filter model positing two cascaded stages of filtering, which fits our data well, and explains observers' ability to segment luminance texture boundary stimuli in the presence of interfering luminance step boundaries. We propose that such computations may be useful for boundary segmentation in natural scenes, where shadows often give rise to luminance step edges which do not correspond to surface boundaries.

## Introduction

**Figure 1:**
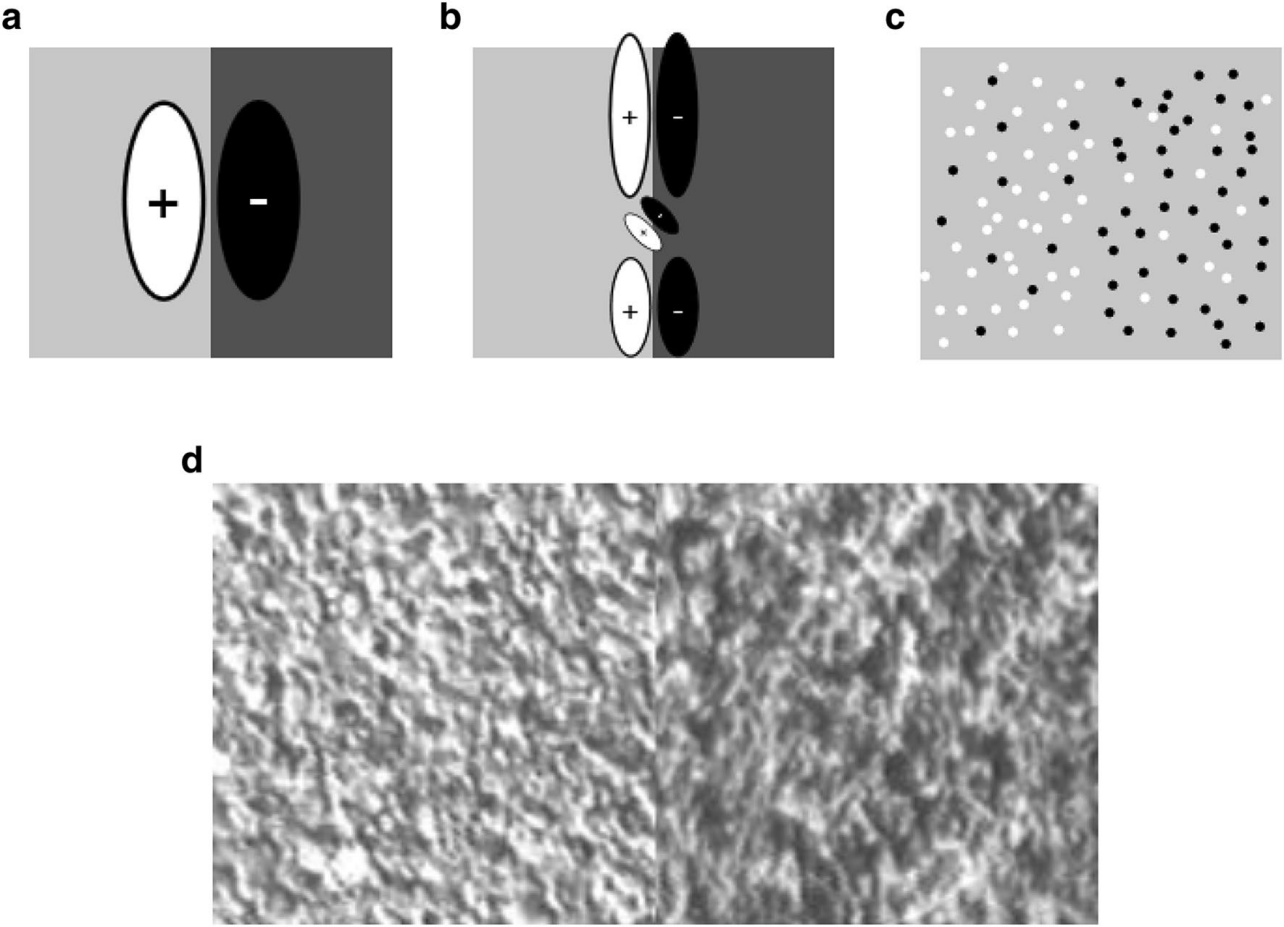
Boundaries without luminance step edges. (**a**) A luminance step boundary (LSB) and a simple detection model in which a linear Gabor filter measures the regional luminance difference. (**b**) Model similar to that in (**a**) where the LSB is analyzed by multiple Gabor filters at varying spatial scales. (**c**) Example of luminance texture boundary (LTB). The luminance difference is defined by differing proportions of black and white micropatterns on each side of the boundary, with no sharp luminance change at the boundary. (**d**) Two juxtaposed textures from the Brodatz database. Although there is clearly a regional difference in luminance, there is no sharp luminance change at the boundary.

Detecting boundaries separating distinct surfaces is a crucial first step for segmenting the visual scene into regions. Since different surfaces generally reflect different proportions of the illuminant, luminance differences provide a highly informative cue for boundary detection in natural images^1–4^. Inspired by physiological findings^5, 6^, a commonly assumed computational model of luminance boundary detection is a Gabor-shaped linear spatial filter of appropriate spatial scale and orientation (or a multi-scale population of filters) detecting a localized change in luminance near the boundary^4, 7^ (Fig. 1a,b). However, in many natural scenes, two distinct surfaces may visibly differ in their mean regional luminance without giving rise to any sharp change in luminance at their boundary. This situation is illustrated in Fig. 1d, which shows two juxtaposed textures from the Brodatz database^8^. Clearly, a large-scale Gabor filter defined on the scale of the whole image as in Fig. 1a can certainly provide some information about a difference in average luminance between the two surfaces. However, it is unknown whether other mechanisms may be better suited to detect regional luminance differences at such boundaries.

In order to address this question, we propose a basic taxonomy of two different ways that luminance cues can define region boundaries. *Luminance step boundaries* (LSBs) are defined by uniform regional differences in luminance, as in Fig. 1a. *Luminance texture boundaries* (LTBs) are defined by differing proportions of dark and light texture elements or micropatterns on two adjacent surfaces (Fig. 1c). Note that for the artificial LTB shown in Fig. 1c there are no textural cues present other than the proportions of dark and light elements on each side of the boundary. Given that regional luminance differences can arise from either LSBs or LTBs, it is of interest to understand whether or not similar mechanisms are employed when segmenting these boundaries, and how LTBs and LSBs interact when both are present, as for example when a cast shadow falls upon a scene region containing one or more surface boundaries.

A number of studies have investigated detection of “first-order” luminance step boundaries^7, 9–11^, as well as detection and segmentation of “second-order” texture boundaries having no luminance difference but differences in texture contrast^12, 13^, density^14^, orientation^15^, polarity^16^ or phase^17^. However, the segmentation of first-order luminance texture boundaries, and the underlying computations, are poorly understood.

In this study, we characterize perceptual segmentation of LTBs (Experiment 1) and demonstrate that simple regional luminance difference computation cannot readily explain their segmentation (Experiments 2, 3). We demonstrate the robustness of LTB segmentation to variations in contrast of texture elements, and demonstrate an excellent fit to the data with a psychometric function incorporating divisive contrast normalization (Experiment 3). We show that when both cues are present, observers can ignore masking LSBs having orthogonal orientations when segmenting LTBs using proportion of imbalanced patterns as a segmentation cue (Experiment 4). However, the presence of a masking LSB having a congruent orientation with the target LTB can in some cases enhance or impair performance (depending on relative phase), suggesting some degree of pre-attentive interaction between cues.

We test the ability of a simple model positing a single stage of filtering which fit the data well in Experiments 2, 3, but it fails to fully explain the results of Experiment 4, suggesting that LTBs and LSBs are segmented by distinct underlying mechanisms. We define and fit a “filter-rectify-filter” (FRF) model positing two stages of filtering to data from Experiment 4, and show that this model successfully accounts for observer performance in the task. Previous studies of second-order vision have fit psychophysical data with FRF models^13, 14, 18^, but here we show that the FRF model can also account for the ability of observers to extract first-order (luminance) information in the presence of masking LSB stimuli. We propose that such mechanisms may be useful for performing boundary segmentation in natural vision, where extraneous stimuli such as shadows often give rise to LSB stimuli which do not correspond to surface boundaries.

## Methods

### Stimuli

#### Luminance texture boundaries

**Figure 2:**
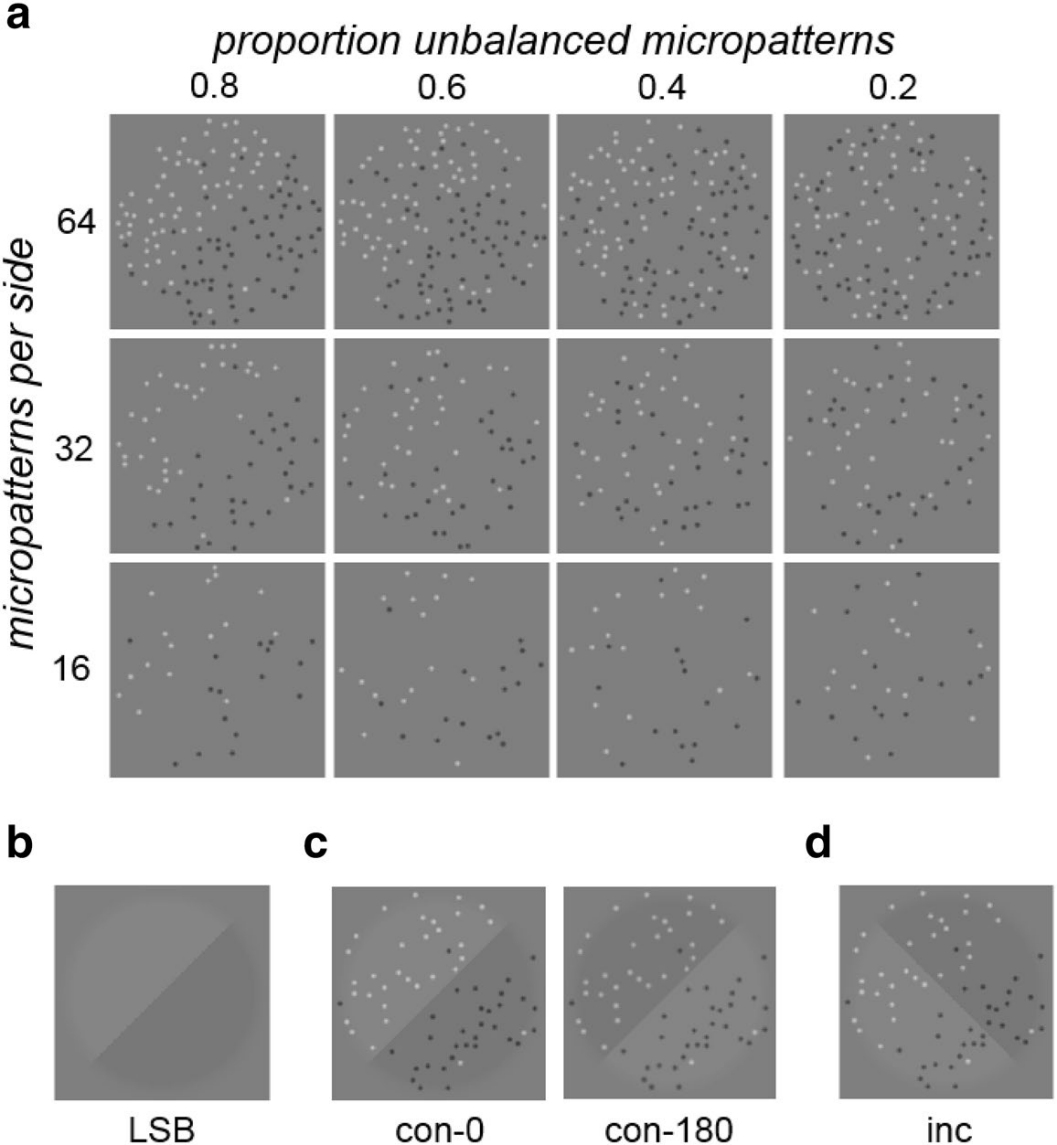
Stimulus images. (**a**) Examples of luminance texture boundary (LTB) stimuli used in this study, shown for varying densities (16, 32, 64 micropatterns on each side of boundary) and proportion unbalanced micropatterns (${\pi }_{U}$ = 0.2, 0.4, 0.6, 0.8). For all of these example stimulus images, the boundary is right oblique. (**b**) Luminance step boundary (LSB) stimulus. (**c**) Stimulus image examples with LTB and LSB having the same orientation (*congruent*), either phase-aligned (**con-0**) or opposite-phase (**con-180**). (**d**) Example image having superimposed, orthogonal (*incongruent*) luminance texture (right-oblique) and luminance step (left-oblique) boundaries (**inc**).

Luminance texture boundary (LTB) stimuli were created by placing different proportions of non-overlapping black (B) and white (W) micropatterns on opposite halves of a circular disc, with the boundary separating the two regions oriented left (L)-oblique (− 45° w.r.t. vertical) or right (R)-oblique (+ 45° w.r.t. vertical), as shown in Fig. 2a. The proportion of black vs. white micropatterns on each side of the LTB was parameterized by the proportion ${\pi }_{U}$ of "unbalanced" micropatterns on each side of the disc (i.e., those not having an opposite side counterpart of the same luminance polarity). Note that ${\pi }_{U}$ can range from 0 (indicating an equal number of black and white micropatterns on both sides) to + 1 (opposite colors on opposite sides).

For the experiments described here, we employed a 256 × 256 pixel stimulus subtending 4° visual angle (dva). An equal number (16, 32 or 64) of non-overlapping micropatterns were pseudo-randomly placed on each side of the boundary, with each micropattern being an 8 pixel Gaussian (σ = 2 pixels). Overlap was prevented by sequential placement of micropatterns during stimulus generation, using an iteratively updated map of locations which already contained micropatterns, making these locations unavailable for new patterns. Unless otherwise specified, the micropattern maximum amplitude $A$ was set to ± 0.25 (W/B) dimensionless luminance units with respect to the gray mid-point (0.5), so these micropatterns were clearly visible. Michelson contrast ${c}_{M}= \left({L}_{max}-{L}_{min}\right)/\left({L}_{max}+{L}_{min}\right)$ of the LTB stimuli is related to the maximum micropattern amplitude $A$ by ${c}_{M}=2A$. In some experiments, we set $A=\pm 0.1$ (roughly 3–4 times LTB contrast detection threshold) to create a more difficult task due to reduced visibility of the micropatterns. The Michelson contrast varies with $A$, but does not vary with ${\pi }_{U}$ or the number of micropatterns (${n}_{p}$). Note that the overall RMS contrast, defined as the ratio of standard deviation to mean intensity, varies with $A$ and ${n}_{p}$, but not with ${\pi }_{U}.$ This is because the overall number of B and W micropatterns are equal and do not vary with ${\pi }_{U}$, which only determines their relative prevalence on opposite sides of the boundary. The B and W micropatterns deviate from the gray mid-point by the same amount, but in opposite directions, so that the overall mean equals the gray mid-point ($0.5$), and the standard deviation (and hence RMS contrast) is proportional to $A\sqrt{{n}_{p}}$.

Stimuli were designed to have zero luminance difference across the diagonal perpendicular to the region boundary (*anti-diagonal*), so that the only available luminance cue was that across the boundary defining the stimulus. For each stimulus we randomized which half of the disc was brighter, which is equivalent to a random 180° rotation of the stimulus. We can also conceptualize which side is brighter as being a function of the phase (0° or 180°) of a periodic modulation of the luminance by an odd-symmetric square wave centered on the boundary.

#### Luminance step boundaries

We also characterized performance on our identification task with luminance step boundary (LSB) stimuli, like that shown in Fig. 2b. LSB stimuli, produced by multiplying an obliquely oriented step edge by a cosine-tapered circular disc, were also 256 × 256 pixels and scaled to subtend 4 dva. The detectability of this edge was varied by manipulating its Michelson contrast ${c}_{M}$, and again which half was brighter (luminance modulation phase) was randomized on each trial.

### Observers

Two groups of observers participated as psychophysical observers in these experiments. The first group consisted of N = 3 observers who were highly experienced with the segmentation tasks. One of these observers was author CJD, and the other two (KNB, ERM) were undergraduate members of the Computational Perception Laboratory who were naïve to the purpose of the experiments. The second group was comprised of N = 13 naïve, inexperienced observers recruited from undergraduate FGCU Psychology classes, as well as N = 1 initially inexperienced lab member. All observers had normal or corrected-to-normal visual acuity. All observers gave informed consent, and all experimental procedures were approved by the FGCU IRB (Protocol number 2014-01), in accordance with the Declaration of Helsinki.

### Visual displays

Stimuli were presented in a dark room on a 1920 × 1080, 120 Hz gamma-corrected Display++ LCD Monitor (Cambridge Research Systems LTD) with mid-point luminance of 100 cd/m^2^. This monitor was driven by an NVIDA GeForce GTX-645 graphics card, and experiments were controlled by a Dell Optiplex 9020 running custom-authored software written in MATLAB making use of Psychtoolbox-3 routines^19–21^. Observers were situated 133 cm from the monitor using a HeadSpot chin-rest.

### Experimental protocols

#### Experiment 1: Segmentation thresholds for LTBs and LSBs

Towards the larger goal of determining whether the two kinds of luminance boundaries (LTB, LSB) are segmented using the same mechanisms, we started by characterizing observers' segmentation thresholds for both kinds of stimulus. In this and subsequent experiments, the psychophysical task was a single-interval classification task, in which the observer classifies a single displayed stimulus as belonging to one of two categories: left- or right- oblique (L/R-oblique).

To study the effects of the number of unbalanced micropatterns on segmentation (Experiment 1a), luminance texture boundaries with 32 micropatterns on each side were presented at nine evenly spaced values of ${\pi }_{U}$ from 0 to 1 in steps of 0.125—example stimulus images are shown in Fig. 2a. A discrete set of values for ${\pi }_{U}$ was necessitated by the need to have a whole number of micropatterns, which was further restricted to be a multiple of 4 to ensure equal numbers of micropatterns in each quadrant. Observers performed 250 psychophysical trials starting at the highest level, with the stimulus level being adjusted using a standard 1-up, 2-down staircase procedure, focusing trials near stimulus levels yielding 70.71% correct responses^22^. Pilot studies with N = 3 experienced observers (CJD, ERM, KNB) showed similar thresholds for 32 and 64 micropatterns, and somewhat higher thresholds for 16 micropatterns (Supplementary Fig. S1), justifying the use of 32 micropatterns as our default micropattern density. LSBs were defined by their Michelson contrast ${c}_{M}$ with respect to the luminance midpoint. LSBs were presented at Michelson contrasts in 11 logarithmic steps from ${c}_{M}=$10^−2.7^ to 10^−1.7^, using the same staircase procedure (Experiment 1b) for 250 trials.

Naïve and inexperienced observers tested in Experiment 1 first obtained experience with segmenting both kinds of boundaries over two training sessions prior to the experiment. During the first training session, they ran two full threshold series for segmenting both LTBs (${\pi }_{U}$ cue) and LSBs (${c}_{M}$ cue). During the second training session, they ran one more series for both cues. Immediately after the second training session, they ran a final (4th) threshold series to estimate stimulus levels for each cue leading to JND (75% correct) performance.

#### Experiment 2: LTBs with constant luminance difference

In order to test the hypothesis that the key variable determining LTB segmentation performance is luminance difference, we generated a series of LTB stimuli having constant luminance difference arising from a fixed number (N = 8) of unbalanced (opposite color) micropatterns on opposite sides of the boundary. By adding an equal number of luminance-balanced micropatterns (i.e. having the same color) to both sides of the boundary (N = 0, 8, 16, 24, 32), we decreased the proportion of unbalanced micropatterns, making the boundary more difficult to segment, while maintaining constant luminance difference across the boundary. Examples of such stimulus images with 0, 16 or 32 additional balanced pairs of micropatterns are illustrated in Fig. 5a.

#### Experiment 3: Segmenting LTBs with varying RMS contrasts

In order to test further whether total luminance difference was a strong predictor of LTB segmentation performance, we repeated Experiment 1 for a single density (32 micropatterns per side) while varying the maximum luminance $A$ of each micropattern with respect to the screen mid-point luminance (0.5). This was accomplished by setting the maximum amplitude of each micropattern to three different levels with respect to the mid-point. W/B micropattern amplitudes were set at *A *= ± 0.1, ± 0.25, ± 0.4 with respect to the luminance mid-point of 0.5 (${c}_{M}=2A= 0.2, 0.5, 0.8$). This had the effect of creating a large range of luminance differences across the LTB, for the same micropattern density.

#### Experiment 4: Segmenting LTBs while ignoring masking LSBs

Of particular interest for the current study is investigating the relationship between the mechanisms used to segment LTBs and those used to segment LSBs. If the mechanisms are fully distinct, an observer should have little difficulty in segmenting a superimposition of an LTB and an LSB (either of the same or different orientations), when instructed to segment using only the LTB cue. Conversely, identical or highly overlapping mechanisms would lead to profound impairment of performance.

To investigate this question, we ran an experiment (Experiment 4a) using author CJD, two naïve experienced observers (EMR, KNB), and N = 6 naïve inexperienced observers. Observers were instructed to segment an LTB target using proportion of unbalanced patterns ${\pi }_{U}$ as the segmentation cue, where ${\pi }_{U}$ was presented at JND (75% correct) as measured for that observer (determined from Experiment 1a). For some trials, a masking LSB (also presented at that observer’s JND), which observers were instructed to ignore, was added to the LTB. There were three kinds of trials in this experiment: 200 *neutral* trials (**neu**) where the LTB was presented in isolation, 200 *congruent* trials (**con**) with the LTB target and masking LSB having congruent boundary orientation (both cues left or right-oblique: see Fig. 2c), and 200 *incongruent* trials (**inc**) with the LTB target and masking LSB having incongruent orientations (one cue left-oblique, the other right-oblique: see Fig. 2d). For the (200) congruent stimuli, in half of trials (100) the two stimuli were consistent in which half had higher luminance (Fig. 2c, *left*), corresponding to luminance modulations that were phase-aligned (**con-0**). For the other half (100) of the trials, they were in conflict (Fig. 2c, *right*), corresponding to luminance modulations that were opposite-phase (**con-180**).

We also performed a second condition of this experiment (Experiment 4b) on three observers (CJD, KNB, MXD) in which the LSB maskers were presented at their JND thresholds determined from preliminary trials in which there was uninformative masking LTB present (${\pi }_{U}=0$, meaning both sides of the boundary had equal numbers of black and white patterns). The JND thresholds in the presence of the uninformative masker were only slightly higher than the JND thresholds for the LSB stimulus in the absence of a masker (~ 1% vs. ~ 0.5%, for these three observers: Supplementary Table S5). Furthermore, even in the presence of the uninformative LTB masker, the lower-contrast (~ 0.5%) LSB used in Experiment 4a was segmented well above chance (~ 60–65% correct).

### Psychometric data analysis

#### Signal-detection theory (SDT) psychometric function

Data was fit using a signal-detection theory (SDT) psychometric function^23, 24^, in which the proportion correct responses (${P}_{C}$) for a single-interval classification task at stimulus level $x$ is given by

1 $${P}_{C}=\Phi \left(\frac{{d}^{^{\prime}}}{2}\right),$$

2 $${d}^{^{\prime}}={\left[gx\right]}^{\tau },$$

where $\Phi$ is the cumulative unit normal distribution and ${d}^{^{\prime}}$ is the separation of the (unit variance) signal and noise distributions. Since stimulus discriminability often varies nonlinearly with reference stimulus intensity^25^, the SDT modeling framework posits that ${d}^{^{\prime}}$ is related to stimulus intensity via a nonlinear *transducer function*^23^. Following this previous study, we utilize the power-law transducer function given in Eq. (2) which has N = 2 free parameters of gain $g$ and transducer exponent $\tau$. The SDT psychometric function was fit to psychophysical data using MATLAB R2016a (http://www.mathworks.com) routines (PAL_SDT_PFML_Fit.m) from the Palamedes Toolbox^24, 26^ (http://www.palamedestoolbox.org/). Data was fit both with and without lapse rates^27, 28^, and nearly identical threshold estimates were observed in both cases, although sometimes fitting without lapse rates under-estimated the psychometric function slope. For the case of the SDT psychometric function fitted using lapse rates,

3 $${P}_{C}=\frac{\lambda }{2}+\left(1-\lambda \right)\Phi \left(\frac{{d}^{^{\prime}}}{2}\right),$$

λ denotes the lapse probability, which was constrained to lie in the range $\left[0, 0.1\right].$ In this case, the SDT psychometric function has N = 3 free parameters.

#### Bootstrapping psychometric functions

For some analyses, bootstrapping^29^ was employed to determine the 95% confidence intervals for both the psychometric function thresholds (Experiment 1), as well as the proportion of correct responses predicted as a function of the stimulus level defined as either ${\pi }_{U}$ or absolute luminance difference (Experiment 3). For bootstrapping analyses, N = 100 or N = 200 simulated datasets were created as follows: for each stimulus level with *n*_*i*_ presentations and *c*_*i*_ experimentally observed correct responses (proportion of correct responses *p*_*i*_ = *c*_*i*_/*n*_*i*_), we sampled from a binomial distribution having *n*_*i*_ trials with probability *p*_*i*_ to create a simulated number of correct responses for that stimulus level. We fit our SDT psychometric function to each of these simulated datasets, to obtain distributions of the psychometric function parameters, as well as the stimulus levels corresponding to JND (75% correct) performance.

### Image-computable models

#### Image-computable SDT model

Models were also fit using one or two derived quantities measured from stimulus images. We refer to such models as image-computable (IC), and this model in particular as IC-SDT. Given stimulus level *x* used to generate the stimulus, we obtained two quantities directly measured from the image: *L*(*x*), which is the absolute value of the difference in luminance across the diagonal corresponding to the target orientation, and *C*(*x*), which is the global RMS stimulus contrast. We then modified the SDT model defined in (1–3) by defining

4 $${d}^{^{\prime}}={\left[g_{1}L(x)\right]}^{\tau_{1} }.$$

In some analyses (Experiment 3), we defined

5 $${d}^{^{\prime}}= \frac{{\left[g_{1}L(x)\right]}^{\tau_{1} }}{{1 + \left[g_{2}C(x)\right]}^{\tau_{2} }}, $$

to model effects of global stimulus contrast *C*(*x*) that might co-vary with luminance differences *L*(*x*) as stimulus level *x* is varied. The form in (5), having N = 4 free parameters (N = 5 with lapse), is only appropriate for experiments in which the global stimulus contrast *C*(*x*) varies, since otherwise it is over-parametrized—in these cases we set *g*_2_ = 0, in which case (5) becomes (4), having N = 2 free parameters (N = 3 with lapse).

#### Image-computable model with one filtering stage

By design of the stimuli used in Experiments 1–3, for each trial image there is no difference in luminance across the anti-diagonal (the axis orthogonal to the stimulus orientation). Therefore, there was no need to take this into account when applying the IC-SDT model (4, 5). However, in the masking experiment (Experiment 4), in the case where the masking LSB has an incongruent orientation, there will be a luminance difference across the anti-diagonal, which can potentially influence the decision. To analyze this data, we apply a more general, image-computable model, which we call IC-1, having N = 2 free parameters. In the IC-1 model, illustrated schematically in Fig. 4a, we assume that each stimulus $x$ gives rise to a decision variable $u\left(x\right)$ which serves as input to the unit normal cumulative density function (CDF) Φ, so that the probability of a “right-oblique” behavioral response ($b=R$) is given by

6 $$P\left(b=R\right)=\Phi \left(u\left(x\right)\right),$$

7 $$u\left( x \right) = \left[ {g_{1} L_{R} \left( x \right)} \right]^{{p_{1} }} - \left[ {g_{1} L_{L} \left( x \right)} \right]^{{p_{1} }} ,$$

where ${L}_{R}(x)$, ${L}_{L}(x)$ are the absolute values of the measured luminance differences across the right- and left-diagonals, ${g}_{1}$ is a gain parameter and ${p}_{1}$ an exponential nonlinearity. The IC-SDT model (1–5) is actually a special case of the IC-1 model (6, 7) in the case of stimuli having zero luminance difference across the anti-diagonal. Therefore, we will refer to both IC-SDT and IC-1 as the “one stage” model (Fig. 4a), although IC-1 is a more general model since it can also account for luminance differences across the anti-diagonal. We also extended the IC-1 model (6, 7) to include divisive normalization by global stimulus contrast $C\left(x\right)$, as in the IC-SDT model (5).

#### Image-computable model with two filtering stages

Masking data from Experiment 4 were fit using a two-stage image-computable model (IC-2), illustrated in Fig. 8a, which first convolves the image with on-center and off-center Difference-of-Gaussians (DOG) filters. The output of this first filtering stage is rectified and then passed to a second stage of filtering which computes a difference in first-stage activity across the left and right oblique diagonals. Second-stage filters were assumed to take a half-disc shape, integrating uniformly across the first stage outputs. The outputs of these second-stage filters are then used (as described below) to calculate a decision variable $u\left(x\right)$. We fixed the first-stage DOG filter properties so that the standard deviation of the Gaussian defining the filter center is matched to the radius of the dots, while that defining the surround has a standard deviation twice that of the center. This choice is consistent with previous classification image studies of Gaussian detection in noise^30^. Mathematically, this filter is defined as

8 $$h\left(x,y\right)=c\left(x,y\right)-{\rho }_{IE}s\left(x,y\right),$$

where $c\left(x,y\right)$ denotes the center, and $s\left(x,y\right)$ the surround, evaluated at $\left(x,y\right)$. The only free parameter for the first stage which we estimate from the data is the ratio ${\rho }_{IE}$ of the amplitudes of the center and surrounds, with ${\rho }_{IE}=0$ indicting no surround. If the rectified luminance differences (with nonlinear exponent ${p}_{1}$) from the left and right ON-center filters is given by ${L}_{L}^{ON}\left(x\right)$, ${L}_{R}^{ON}\left(x\right)$, and from the OFF-center filters ${L}_{L}^{OFF}\left(x\right)$, ${L}_{R}^{OFF}\left(x\right)$, our decision variable is

9 $$u\left(x\right)={\left[{g}_{2}{L}_{R}^{ON}\left(x\right)\right]}^{{p}_{2}}+{\left[{g}_{2}{L}_{R}^{OFF}\left(x\right)\right]}^{{p}_{2}}-{\left[{g}_{2}{L}_{L}^{ON}\left(x\right)\right]}^{{p}_{2}}-{\left[{g}_{2}{L}_{L}^{OFF}\left(x\right)\right]}^{{p}_{2}},$$

where ${g}_{2}$, ${p}_{2}$ are gains and nonlinearities for the second-stage filters. The two-stage model (IC-2) only contains N = 4 free parameters (${\rho }_{IE}$, ${p}_{1}$, ${p}_{2}$, ${g}_{2}$) which we estimate by fitting to data. To make computations tractable, we pre-filtered the stimuli with the center-surround DOG filters with IE amplitude ratios given by ${\rho }_{IE}= 0.1, 0.15, 0.2, 0.25, 0.3, 0.35, 0.4$ and then optimized (MATLAB fmincon.m) the remaining parameters for each value of ${\rho }_{IE}$. A DC-balanced filter (zero response to constant illumination) is obtained for ${\rho }_{IE}\approx 0.3$ (2.996). For smaller values the filter has a net excitatory receptive field, and for larger values it is net inhibitory. Two observers (CJD, JCO) had optimal ${\rho }_{IE}$ values at one end-point of our search range (${\rho }_{IE}$ = 0.1), whereas all other observers had optimal ${\rho }_{IE}$ values between 0.15 and 0.3. Therefore, for these two observers the search range was expanded to include ${\rho }_{IE}= 0.0, 0.05$ to find the optimal value. Initial starting points for the optimization were found using a 3-D grid search with ${p}_{1}$, ${p}_{2}$ taking grid values [0.5, 1, 2] and ${g}_{2}$ taking grid values from 10^−3^ to 10^1^ in 5 log steps.

### Model comparison

To evaluate the relative goodness-of-fit of different models, we make use of the Bayes Information Criterion (BIC), which is an asymptotic approximation to the log-posterior density^31, 32^, given by the formula

10 $${\text{BIC }}\equiv {\text{ln}}P\left(M|D\right)\cong {\text{ln}}L\left(D|M\right)-\frac{K}{2}{\text{ln}}n,$$

where $L\left(D|M\right)$ is the data (*D*) likelihood given the fitted model (*M*), *K* is the number of free model parameters, and *n* is the number of data points. Since exponentiating (10) yields the posterior probability (up to a constant), larger values of the BIC indicate a better fit. Sometimes the BIC is defined by multiplying (10) by a factor of − 2, so that a smaller value indicates a better fit^33^. Using the definition in (10), the posterior probability ratio for two models can be obtained by simply exponentiating the difference of their respective BICs (e.g. Eq. (10) of Reference^33^).

## Results

### Luminance texture boundary stimuli

In order to quantitatively examine the segmentation of luminance texture boundaries (LTBs), we defined a set of LTB stimuli which allowed us to vary the luminance across a boundary by varying the proportion of black and white micropatterns within in each region (Fig. 2a). When there are equal numbers of black (B) and white (W) micropatterns on each side of the boundary, each micropattern is *balanced* by another of the same color on the other side. In this case, the luminance difference between regions is zero. When one side has more W patterns, and the opposite side has more B patterns, a proportion of the patterns on each side are *imbalanced*, giving rise to a difference in luminance across the diagonal. Therefore, we can modulate the luminance difference and therefore the boundary salience by changing the proportion of patterns on each side that are unbalanced (${\pi }_{U}$), as illustrated in Fig. 2a. A value of ${\pi }_{U}=0$ corresponds to no boundary, whereas ${\pi }_{U}=1$ means that all the patterns on each side are the same.

### Experiment 1: Measuring segmentation thresholds

**Figure 3:**
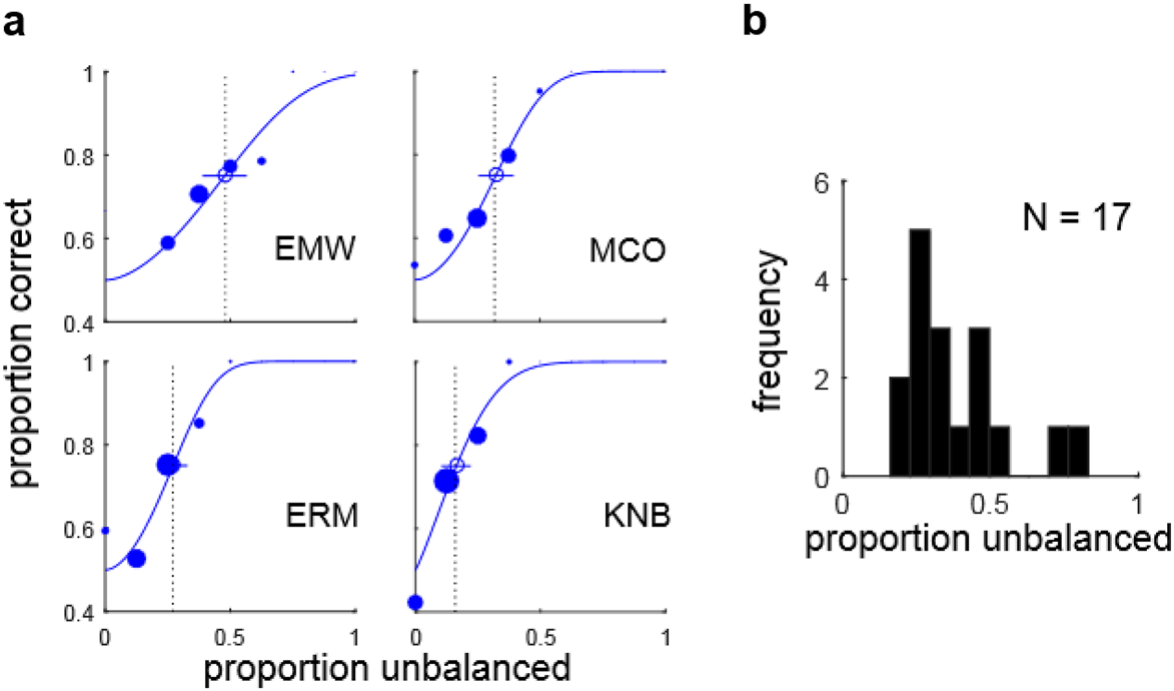
Psychometric functions and threshold distributions. (**a**) Psychometric functions and fitted functions based on SDT model (blue curves) for four observers (EMW, MCO, ERM, KNB) performing luminance texture boundary (LTB) segmentation (Experiment 1a) as a function of the proportion unbalanced micropatterns (${\pi }_{U}$), i.e. the proportion of micropatterns not having a same-polarity counterpart on the opposite side of the boundary. The size of each solid dot is proportional to the number of trials obtained at that level, and dashed black lines denote 75% thresholds for the fitted curves. Circles and lines indicate threshold estimates and 95% confidence intervals obtained from 200 bootstrapped re-samplings of the data. (**b**) Histogram of segmentation thresholds (${\pi }_{U}$) measured from all observers (N = 17) in Experiment 1a.

In Experiment 1a, we examined the ability of observers to segment LTBs using the proportion of unbalanced micropatterns (${\pi }_{U}$) as a cue. Figure 3a shows the SDT psychometric function (2, 3) fit to data from two representative inexperienced observers (EMW, MCO) and two experienced observers (ERM, KNB). Nearly identical threshold estimates were obtained with and without lapse rates (Supplementary Fig. S2a). A histogram of JND thresholds (75% correct) for all observers is shown in Fig. 3b. The median observer could perform the task with a threshold of ${\pi }_{U}=0.31$, and the best observer could reliably segment at ${\pi }_{U}=0.16$, suggesting a strong sensitivity to the proportion of unbalanced micropatterns on the two surfaces. In Experiment 1b we also determined LSB segmentation thresholds for luminance disc stimuli like that shown in Fig. 2b in units of Michelson contrast for the same observers tested in Experiment 1a (Supplementary Fig. S3).

### Evaluating a simple model

**Figure 4:**
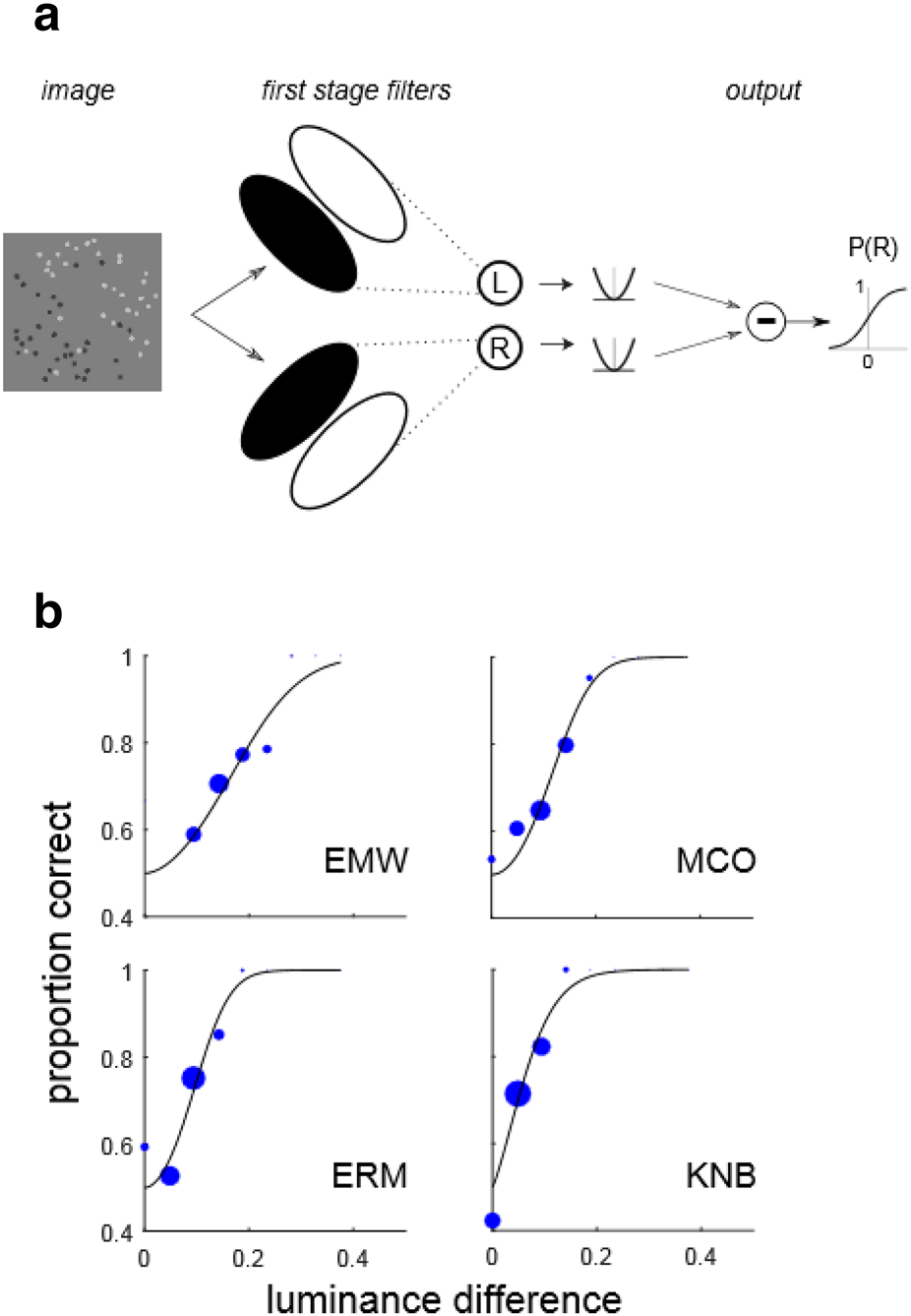
Single-stage filter model. (**a**) Image-computable model with a single stage of filtering (IC-1). Luminance differences are computed across the left-oblique and right-oblique diagonals, passed through a rectifying, exponentiating nonlinearity and subtracted to determine the probability P(R) of observer classifying the boundary as right-oblique. In the case where there is only a luminance difference across one diagonal, this model is equivalent to the IC-SDT model (Eq. (4)). (**b**) Fits of the model in (a) to LTB segmentation data from Experiment 1a for the same observers as in Fig. 3a.

One simple explanation for LTB segmentation performance is that the visual system is performing a simple luminance difference computation, similar to that illustrated schematically in Fig. 4a. As the proportion of unbalanced micropatterns increases, so does this luminance difference, making the LTB more visible. We implemented an image-computable model derived from the SDT psychometric function (IC-SDT, Eq. (4)) in which performance was a function of the stimulus-level dependent luminance difference $L\left(x\right)$ across the diagonal boundary. We see in Fig. 4b that this simple model predicts observer performance quite well as function of the luminance difference for LTB stimuli. Likewise, this model predicts performance well for LSB stimuli (Supplementary Fig. S4).

### Experiment 2: Holding luminance difference constant

**Figure 5:**
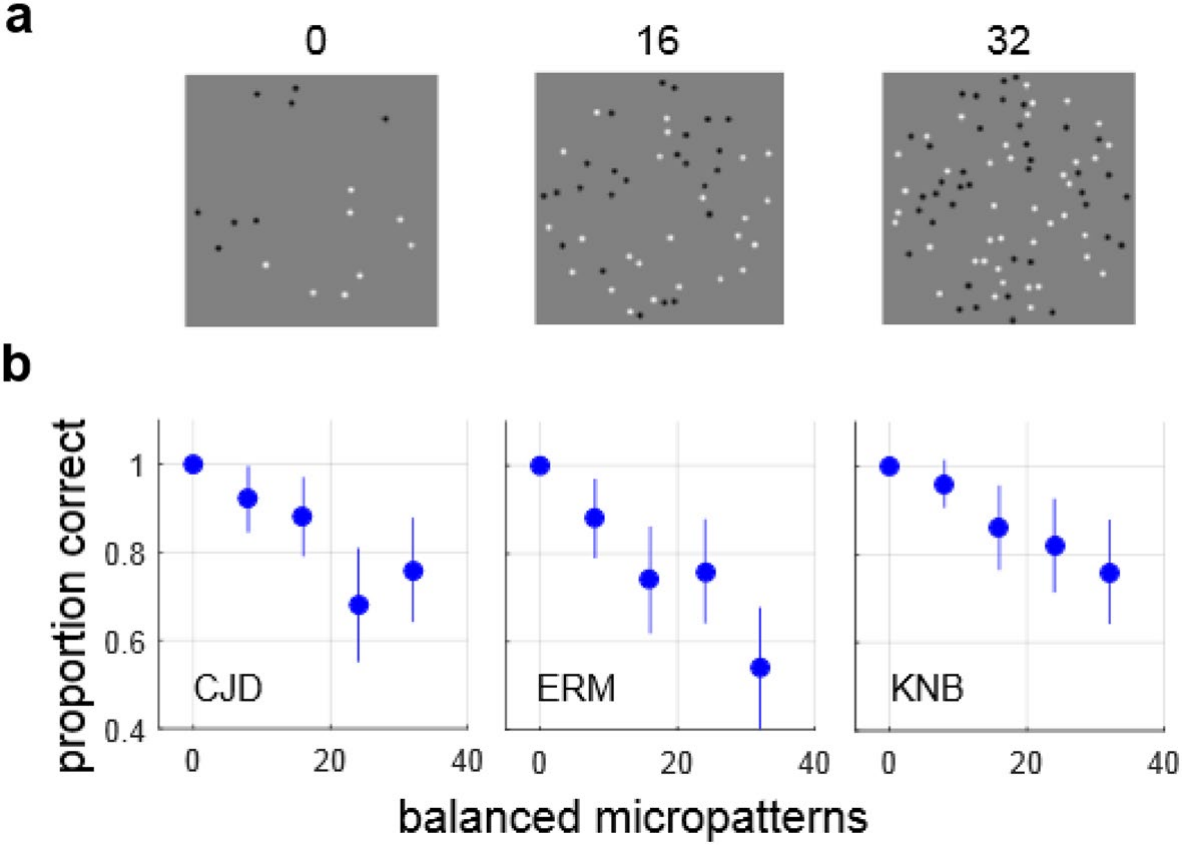
Holding luminance difference constant. (**a**) Examples of LTB stimuli used in Experiment 2, having an equal number (8) of unbalanced micropatterns on each side of the boundary, with varying numbers (0, 16, 32) of balanced micro-patterns. In this series, the luminance difference across the boundary is constant for all stimuli. (**b**) Proportion correct responses for three observers for differing numbers of balanced micropatterns. Lines indicate 95% binomial proportion confidence intervals for each level (N = 50 trials at each level). We see that performance degrades significantly with increasing numbers of balanced micropatterns, despite constant luminance difference. This suggests that a simple luminance difference computation may be inadequate to explain segmentation of LTB stimuli.

In order to directly test whether a simple luminance difference computation like that shown in Fig. 4a is adequate to explain LTB segmentation, in Experiment 2 we constructed a series of LTB stimuli having an identical number of unbalanced micropatterns on each side, which provide the segmentation cue, while increasing the number of balanced patterns on each side, which serve as distractors. Stimuli from this experiment are illustrated in Fig. 5a. We see in Fig. 5b that for all three observers tested, performance decreases as the number of distractors increases, with all observers showing a significant effect of the number of distractors (Pearson’s chi-squared test; CJD: χ^2^(4) = 25.32, *p* < 0.001, ERM: χ^2^(4) = 34.817, *p* < 0.001, KNB: χ^2^(4) = 18.56, *p* = 0.001). These results argue against the hypothesis that LTB stimuli are segmented using a simple luminance difference computation, at least in cases like this where the total number of micropatterns co-varies with the proportion of unbalanced patterns.

### Experiment 3: Varying contrast while segmenting by proportion unbalanced patterns

**Figure 6:**
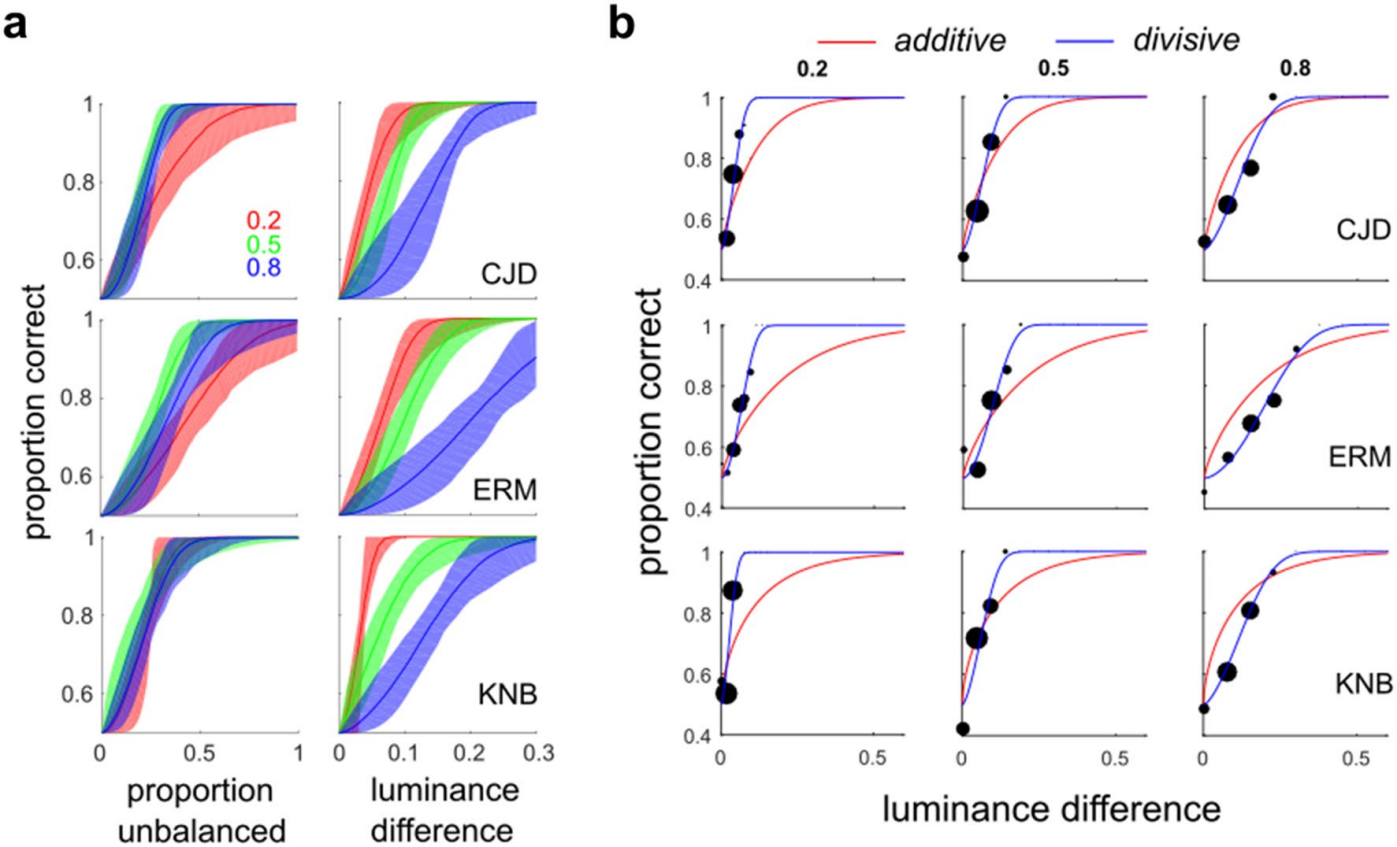
Using micro-pattern amplitude to vary global luminance difference. (**a**) Bootstrapped SDT psychometric function fits (200 bootstrapped re-samplings) with 90% confidence intervals of observer performance as a function of proportion unbalanced micropatterns (left panels) and absolute luminance difference (right panels). This shows that identical luminance differences give rise to significantly different levels of observer performance for the three Michaelson contrasts (right panels), i.e. global luminance difference is a very poor predictor of performance. Instead, observer performance is much better predicted by the proportion of unbalanced micro-patterns, (almost) irrespective of micro-pattern amplitude (left panels). (**b**) Data from Experiment 3 (black dots) and fits of the additive (red) and divisive (blue) image-computable signal detection theory models (IC-SDT) to the data. Each observer was tested at three different maximum micro-pattern amplitudes, which correspond to different Michaelson contrasts (0.2, 0.4, 0.8) of the stimuli. We see that a model incorporating a global luminance difference computation followed by contrast normalization (blue) provides an excellent fit to this data.

As suggested by Experiment 2, a simple luminance difference computation is not a plausible candidate for segmenting LTB stimuli. In Experiment 3, we adduce additional evidence against this simplistic model. In this experiment, three observers (CJD, KNB, ERM) segmented LTB stimuli using the proportion of unbalanced micropatterns ${\pi }_{U}$ as a cue, as in Experiment 1a. This was performed for three different levels of the stimulus Michelson contrast (${c}_{M}=0.2, 0.5, 0.8$). This had the effect of creating drastically different regional luminance differences for stimuli in different series having the same proportion of unbalanced micropatterns ${\pi }_{U}$. As we see in Fig. 6a, ${\pi }_{U}$ (left panels) is a much better predictor of observer performance than the absolute luminance difference (right panels). Therefore, despite wide variation in the absolute difference in luminance across the boundary at different contrasts, observers are still able to segment boundaries differing in the proportion of light and dark areas in the two regions.

### Extending the one-stage model: divisive computations

One can account for observer performance in Experiments 2 and 3 using a single-stage model like that in Fig. 4a by introducing a contrast normalization operation (5). Using data from all three contrast levels in Experiment 3, we fit both the standard IC-SDT model using simple luminance difference only (4), as well as the divisive IC-SDT model incorporating both luminance difference and RMS contrast normalization (5). As we see in Fig. 6b, the fit of the standard additive SDT model (red lines) is quite poor compared to the divisive IC-SDT model (blue lines). Since the divisive model has more parameters, we compare the goodness-of-fit using the Bayes Information Criterion (BIC), which rewards goodness of fit while penalizing model complexity^31–33^. The BIC analysis suggests a strong preference^34^ for the divisive model for all observers (Supplementary Table S1). Similar results were obtained using models with lapse rates estimated as well (Supplementary Fig. S5a). In addition, we see that the divisive model is able to do a reasonably good job of predicting observer performance in Experiment 2 (Supplementary Fig. S5b, *red symbols*).

### Experiment 4: Segmenting LTBs while ignoring LSBs

The results of Experiments 1–3 suggest that a model implementing a luminance difference computation (Fig. 4a) with contrast normalization can potentially explain LTB segmentation performance. However, one weakness of a single-stage model computing simple luminance differences is that it may be susceptible to interference from masking LSBs having incongruent orientations. Motivated by these considerations, in Experiment 4 we investigated the extent to which segmentation of LTB stimuli is influenced by the presence of masking LSB stimuli which observers are instructed to ignore. The logic of this paradigm is that if LTBs and LSBs are processed by entirely different mechanisms, then the presence of a task-irrelevant LSB should have no effect on segmentation using the LTB cue. If one cue cannot be ignored, it suggests that there may be some overlap or interaction between the mechanisms. This sort of paradigm was used in a previous study^35^ to demonstrate that second-order color and texture cues were not processed independently.

In Experiment 4a, N = 9 observers segmented LTB stimuli as in Experiment 1a using proportion of unbalanced micropatterns as a cue, with ${\pi }_{U}$ set to the observer’s 75% performance threshold. For 200 *neutral* trials, the LTB was presented in isolation, for 200 *congruent* trials a masking LSB at segmentation threshold was presented with the same orientation (L/R oblique) as the target (Fig. 2c), and for 200 *incongruent* trials the LSB was presented at the orthogonal orientation (Fig. 2d). For half of the congruent trials, the LTB and LSB were phase-aligned (Fig. 2c, "**con-0**", left), and for the other half they were opposite-phase (Fig. 2c, "**con-180**", right).

**Figure 7:**
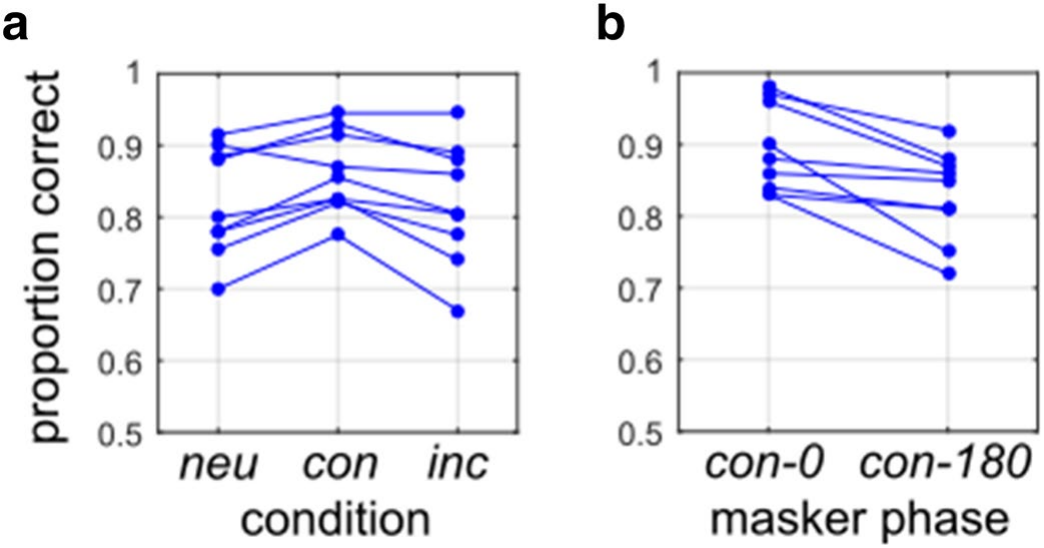
Effects of masking LSBs on LTB segmentation. (**a**) Performance for N = 9 observers in Experiment 4a, segmenting LTB stimuli using a proportion of unbalanced micro-patterns (${\pi }_{U}$), set at 75% JND for each observer, as measured in Experiment 1a. We see similar performance for most observers in the absence of a masker (neutral case, **neu**) as well as with a masker having congruent (**con**) and incongruent (**inc**) orientation. Here the congruent case pools across in-phase and opposite-phase conditions. (**b**) Performance for same observers for congruent stimuli which are in-phase (**con-0**) and opposite-phase (**con-180**).

As we can see from Fig. 7a, performance when segmenting LTB stimuli when using ${\pi }_{U}$ as the cue is quite robust to interference from masking LSB stimuli. Statistical tests (Pearson’s Chi-squared) comparing observer performance across all three conditions did not find any significant effect of condition (*neutral* (**neu**), *congruent* (**con**), *incongruent* (**inc**)) for any individual observer (Supplementary Table S2). Pooling across all observers, we did however obtain significantly different (**χ**^**2**^(2) = 15.319, *p* < 0.001) values of proportion correct for each condition (**neu**: 0.8217, **con**: 0.8622, **inc**: 0.8189), due to slightly enhanced performance for congruent masking LSBs, since there was no impairment for incongruent masking LSBs (**χ**^**2**^(1) = 0.047, *p* = 0.828).

The enhanced performance for congruent masking LSBs was phase-dependent, as seen in Fig. 7b. For the aligned-phase case (**con-0**), we observe significant improvements in performance over the neutral condition for 4/9 observers (Supplementary Table S3). We fail to find any significant difference in individual observer's performance between the neutral and opposite-phase (**con-180**) cases. Pooling across observers, we find significant differences (**χ**^**2**^(1) = 24.383, *p* < 0.001) between the proportions correct for the neutral case and the aligned-phase case (**neu**: 0.8217, **con-0**: 0.8944). However, we fail to find a significant difference (**χ**^**2**^(1) = 0.288, *p* = 0.592) between the proportion correct in the neutral case and the opposite-phase case (**con-180**: 0.8300). In at least some observers (3/9 total, 2/8 naive) we see improved performance for phase-aligned compared to opposite-phase boundaries in the congruent case (Fig. 7b, Supplementary Table S4), as well as a significant effect (**χ**^**2**^(1) = 15.732, *p* < 0.001) pooling across all observers (**con-0**: 0.8944, **con-180**: 0.8300).

As a control, we ran another condition (Experiment 4b) on three observers (CJD, KNB, MXD) in which the masking LSB was presented at its JND measured in the presence of a non-informative LTB (${\pi }_{U}=0$). This JND was found to be slightly higher than the JND obtained for these same observers in Experiment 1a (~ 1% vs. ~ 0.5% Michelson contrast, Supplementary Table S5). However, despite this stronger LSB masker, the results from Experiment 4b were qualitatively identical to those in Experiment 4a (Supplementary Fig. S8a,b). Two out of three (2/3) individual observers (KNB, MXD) did not demonstrate any significant effect of the congruency condition (Supplementary Table S6), or any significant difference between the neutral case and the two congruent phase-alignment conditions (Supplementary Table S7). Only author CJD showed significant effects, exhibiting interference (relative to **neu**) in the **inc** and **con-180** cases, and facilitation in the **con-0** case. This was qualitatively consistent with CJD’s results in Experiment 4a (Supplementary Tables S2, S3). For the two congruent conditions, observers CJD and MXD both demonstrated significant differences between the **con-0** and **con-180** phase conditions (Supplementary Table S8), also consistent with Experiment 4a.

### Evaluating one-stage and two-stage models

**Figure 8:**
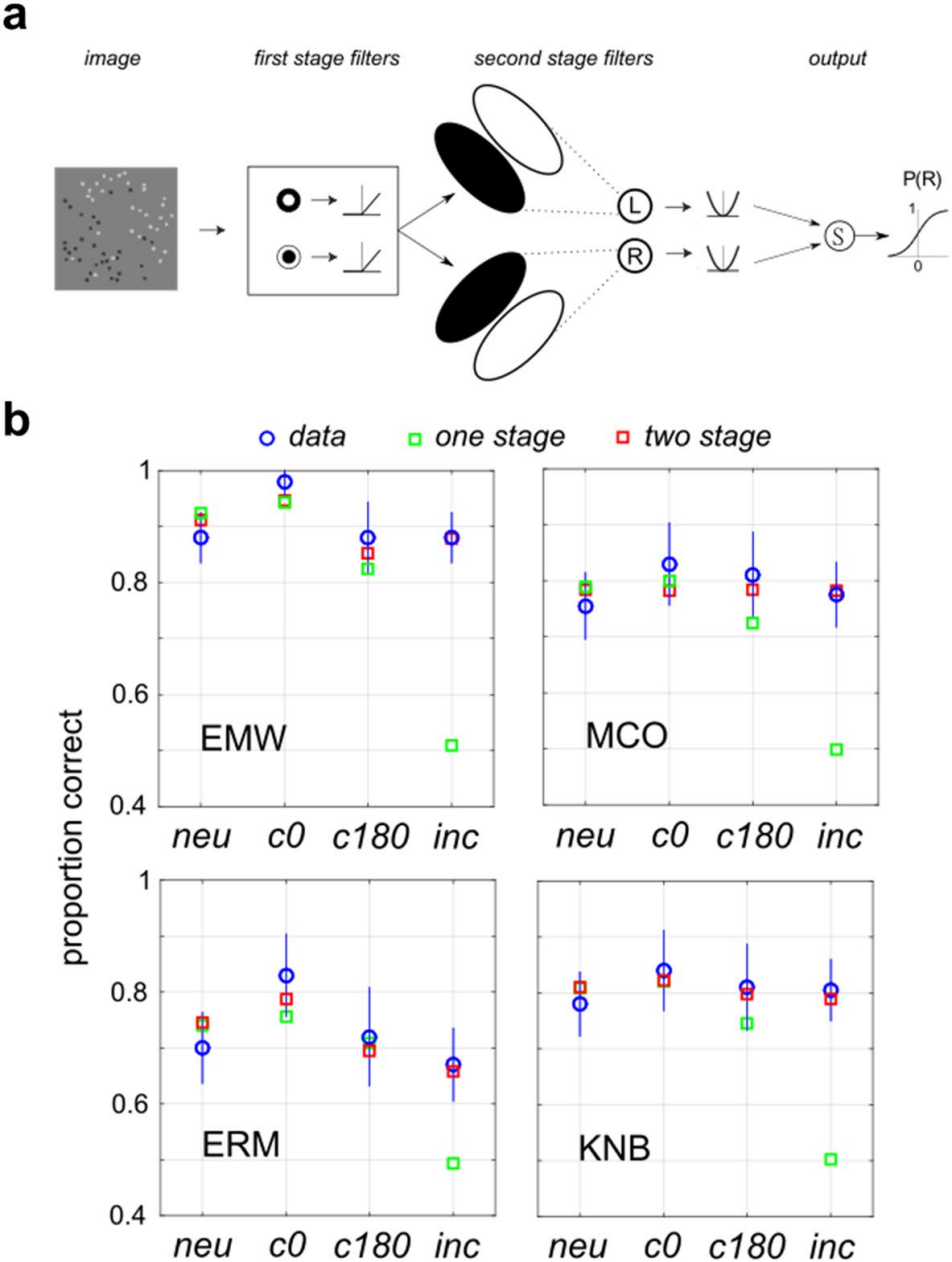
Two-stage model fits Experiment 4 results. (**a**) Model with two cascaded stages of filtering (IC-2). The first stage of this model detects texture elements (here, micro-patterns) on a fine spatial scale. The second stage looks for differences in the outputs of these first-stage filters on the coarse spatial scale of the texture boundary, at either of two possible orientations. Such a model can detect differences in the proportions of black and white micro-patterns on opposite sides of the boundary, while being fairly robust to interference from luminance steps. (**b**) Fits of single-stage model IC-1 (green squares) and two-stage model IC-2 (red squares) to data from Experiment 4a (blue circles, lines denote 95% confidence intervals), for four ways of combining LTB and LSB stimuli: neutral (**neu**); congruent, in-phase (**c0**); congruent, opposite phase (**c180**); and incongruent (**inc**).

Given our findings that LTB segmentation is fairly robust to interference from masking LSB stimuli, it seemed likely that LTBs might be detected by a distinct mechanism. Consequently, we considered the possibility that LTB segmentation may be better explained by a model like that shown in Fig. 8a with two stages of processing (IC-2), rather than a single stage as in the model in Fig. 4a. The first stage is comprised of small-scale spatial filters, implemented here as center-surround filters (see “Methods”, Eq. (8)) which are convolved with the input image and whose outputs are passed through a rectifying nonlinearity. The second stage analyzes the first-stage outputs, with two large-scale filters selective for left-oblique and right-oblique boundaries. These second-stage filter outputs are rectified, exponentiated, and subtracted to determine the probability of an “R” response (“Methods”, Eq. (9)). Note that since the center-surround filters in the first stage are poorly driven by constant light levels, this model can in principle exhibit robustness to interference from LSBs, while still permitting some degree of influence, depending on the relative strengths of the center-surround units, which determines the response of the filter to mean luminance.

Since in Experiment 4 there can be luminance differences across both the diagonal as well as the anti-diagonal, it was necessary to use a more general one-stage image-computable model than the IC-SDT model (4, 5) applied in Experiments 1–3. Therefore, we applied a more general model (IC-1, Eqs. (6), (7)) which compares luminance differences across the left and right diagonals to form a decision variable (See “Methods”).

Figure 8b shows the fits of both the one-stage model IC-1 (Fig. 4a) and two stage model IC-2 (Fig. 8a) to data obtained from Experiment 4a for four observers (EMW, MCO, ERM, KNB). One stage models were fit both with and without divisive normalization terms, and identical predictions of observer performance were obtained. We see in Fig. 8b that although both one-stage (green squares) and two-stage (red squares) models fit observer performance (blue circles) in the neutral (**neu**) and two congruent cases, the one-stage model clearly fails to account for observer performance in the incongruent case (**inc**), predicting near-chance performance. Plots like those in Fig. 8b are shown for all other observers in Supplementary Fig. S6. The lack of robustness of the one-stage model to incongruently oriented LSBs argues strongly in favor of the two-stage model as a more plausible mechanism for LTB segmentation, at least in the presence of interfering LSBs. We observed a strong preference for the two-stage model for all observers in Experiment 4a, as measured by the BIC (Supplementary Table S9).

As shown in Fig. 8b, for the majority of observers, we obtain better LTB segmentation performance in the presence of a congruent boundary with aligned phase (**con-0**) than opposite phase (**con-180**). This difference is also evident for some of the other observers (Supplementary Fig. S6). Interestingly, the two-stage model allows for LSB stimuli to potentially influence LTB segmentation via a center-surround imbalance of the first-stage filters which can provide a mean-luminance ("DC") response. That is, if the on-center (off-center) filters have a small positive (negative) response to constant light levels, this would allow LSB stimuli to exert an excitatory influence on the second-stage filters, potentially explaining the slightly improved performance for the phase-aligned vs. opposite-phase congruent case in Experiment 4 (Fig. 7b). We found that for 6/9 observers in Experiment 4a, the first-stage linear filters had a positive DC response, whereas for 3/9 it was approximately to zero (Supplementary Table S9).

We investigate whether the two-stage model in Fig. 8a can also account for the results of Experiment 3 (Fig. 6). We find that as with the one-stage model, an excellent fit to the data (blue lines) is obtained using the two-stage model when a divisive normalization term is included (Supplementary Fig. S7).

Model fits to the data obtained in Experiment 4b yielded identical results to the fits obtained from Experiment 4a (Supplementary Fig. S8c). For each individual observer we see a strong preference of the BIC measure for the two-stage model (Supplementary Table S10), consistent with Experiment 4a. We found a positive DC response for the first-stage filters for each observer, also consistent with our results in Experiment 4a (Supplementary Table S10).

## Discussion

### Luminance textures

Over half a century of research in modern vision science has investigated visual texture segmentation using parametric stimuli^36–38^. However, this psychophysical work has largely focused on manipulating changes in second-order or higher-order statistical properties which can define texture boundaries, while holding first-order (luminance) cues constant^14, 18^. This is a sensible research strategy because it neatly isolates the problem of understanding how higher-order statistics influence segmentation. However, it is ultimately incomplete since natural region boundaries typically contain first-order cues like color and luminance^1, 39–41^, which can combine with higher-order cues for localization and segmentation ^2, 40, 42–44^. In most studies in which first-order cues are manipulated, they are presented as steps or gratings^7, 9^—or when they are measured from natural images, it is as average luminance within a region^2, 40^. However, as we see in Fig. 1, differences in mean luminance can also be caused by differences in the proportion of light and dark pixels in each surface region, with no abrupt change in albedo at the boundary. We refer to boundaries of this kind as luminance texture boundaries (LTBs), to distinguish them from luminance step boundaries (LSBs). Understanding whether or not these two kinds of luminance cue (LTB, LSB) are processed via the same, different, or partially overlapping mechanisms is of great utility for understanding how first-order and higher-order cues combine to enable natural boundary segmentation. The present study provides a first step in this direction, suggesting that multiple mechanisms may contribute to luminance-based boundary segmentation in natural vision.

### Multiple mechanisms for segmentation using luminance cues

Clearly, whenever there are mean differences in luminance between two regions, a single stage of linear filtering (Fig. 4a) is capable of detecting this difference, for both LTBs (Fig. 4b) and LSBs alike. However, this simplistic model would make the prediction that for any two boundaries with equal luminance differences, segmentation performance should be identical. Explicitly testing this idea in Experiment 2 and Experiment 3 lead us to reject this model. Further exploration revealed that we can however explain the LTB segmentation data in Experiments 2, 3 with a single stage of linear filtering if we incorporate a divisive operation^45^ which normalizes filter outputs by global RMS contrast. Nevertheless, even with this improvement, any model positing a single filtering stage that computes a luminance difference is highly susceptible to interference from stimuli which provide extraneous luminance cues, for instance a cast shadow edge (LSB) with an orientation conflicting with the LTB orientation. We test this prediction explicitly in Experiment 4, where we investigated the ability of observers to segment LTB stimuli in the presence of masking LSB stimuli. In this experiment, we find that LTB segmentation is remarkably robust to interference from masking LSB stimuli. This robustness to masking argues against the idea that a single stage of filtering is adequate to fully explain LTB segmentation.

We posit that two sequential stages of filtering on different spatial scales may be required to explain LTB segmentation, and consider the two-stage model shown in Fig. 8a. It is comprised of an initial layer of filtering on a local spatial scale which detects the texture elements (micropatterns), followed by a second-stage of filtering which looks for spatial differences in the rectified outputs of the first-stage filters on a global scale. This model successfully explains the ability of observers to segment LTB stimuli in the presence of masking LSBs (Fig. 8b), and also accounts for the LTB segmentation data obtained in Experiment 3 (Supplementary Fig. S7). Although the first stage filters in our model are implemented as center-surround filters, which are known to be present in area V1^46, 47^, orientation-tuned mechanisms pooled across different orientations can in principle serve the same function^16^. This general model architecture is known as a Filter–Rectify–Filter model^48^, and has been applied in dozens of studies to model texture segmentation and second-order vision^49^. To our knowledge, the present study is the first time that it has been explicitly demonstrated that an FRF-style model can describe how observers segment textures defined entirely by first-order luminance cues.

One important finding from this study is that although LTB segmentation is highly robust to interference from masking LSB stimuli, it is not entirely independent. For instance, in Experiment 4 we found that segmentation performance was slightly better for a congruently aligned superposition of LTB and LSB when they were phase-aligned compared to when in opposite phase (Fig. 7b). This interaction between LTB and LSB cues could arise in one of two possible ways. One possibility, suggested by our model fitting, is that the first-stage filters are not zero-balanced, so that they would exhibit a significant response to mean luminance. In particular, we observed that the on-center filters which best fit the data from Experiment 4 had a slightly positive response to a constant uniform stimulus (Supplementary Tables S9, S10). This residual luminance response of first-stage filters is consistent with previous psychophysical studies^30^, as well as known neurophysiology of center-surround retinal ganglion cells^50^. This idea also has some similarity to a recently proposed model for how visual cortex neuronal responses to contrast modulation and luminance stimuli might arise from Y-type retinal ganglion cells that are driven by non-zero-balanced bipolar cells^51^. However, another possibility is that the final decision arises by integrating the outputs of a two-stage model like that in Fig. 8a containing zero-balanced filters, with the outputs of a single-stage model like that in Fig. 4a. Such a model would also be consistent with our observations, and it is of interest for future work to design an experiment which could distinguish between these two possibilities.

### Future directions

Although natural surfaces may have luminance differences which arise due to luminance texture boundaries, many other textural differences do not involve changes in luminance. Micropattern orientation, density, contrast and other image features might all provide powerful segmentation cues^12–16, 18^, which may be combined with luminance (and chromatic) cues to enable segmentation in natural vision. A number of previous studies have addressed the issue of first- and second-order cue combination in the special context of contrast modulation^52–55^. More recent work has demonstrated that in-phase luminance and contrast modulations can accompany changes in surface illumination, and integrating these cues provides information important for shape-from-shading^56, 57^. In general it is of great interest for future research to understand how luminance textures combine with second-order cues for segmentation, since this situation probably occurs frequently in natural images. Slight modifications to the stimuli utilized here would permit us to address these questions. For instance, we could define the black and white micropatterns as oriented bars or Gabors instead of dots, and simultaneously manipulate orientation and luminance cues to see how they summate, e.g. via probability summation or additive summation^23^.

Another important issue not addressed in the current study is the relative importance of the black and white micropatterns for segmenting LTBs. A number of psychophysical studies have demonstrated that human observers detect light decrements better and faster than light increments^58–61^. Perhaps consistent with this, neurophysiological studies have revealed that "OFF cells" (responding more strongly to light decrements or to dark stimuli) are more prevalent than ON cells in V1^62, 63^, and that OFF cells support faster processing^60^ and higher spatial resolution^64^. It would be of great interest to apply psychophysical system identification^13^ to fit more sophisticated versions of the model in Fig. 8a to a larger psychophysical dataset in order to determine if greater weight is applied to the OFF pathway in our task, and to what extent this might depend on contrast^65^.

The present study suggests the possibility of neural mechanisms tuned to LTBs which are minimally influenced by overlapping LSBs. We hypothesize that individual neurons tuned to LTBs will most likely be found in extra-striate areas, for instance V2, which contains neurons sensitive to second-order boundaries^66, 67^ and V4, in which some neurons exhibit texture selectivity^68, 69^. As suggested by our psychophysical models, neurons at higher areas of the visual pathway may receive inputs from neurons in V1 or V2 responsive to the micropatterns or texture elements. If the afferent presynaptic V1 neurons in one spatial region are optimally driven by light micropatterns, and those in an adjacent spatial region prefer dark micropatterns, the downstream extrastriate neuron might then be sensitive to differences in the proportion in light and dark micropatterns in these adjacent regions. It is of great interest for future neurophysiology studies to see if neurons can be observed which are selectively responsive to LTB stimuli, while being poorly driven, if at all, by step edges. Such neurons could provide a physiological basis for the ability to segment surface boundaries in the presence of shadows and distinguish shadow edges from boundaries^41, 70^.

Finally, a large body of work has demonstrated that deep neural networks trained on visual tasks like object recognition develop intermediate-layer representations which are sensitive to textural features^71–73^. It would be of great interest for future investigation to study deep neural networks resembling the ventral visual stream^72^ in order to look for neurons which are tuned to luminance texture boundaries while being relatively unresponsive to luminance steps, and to see if decoding such a population of units could account for human psychophysical performance in texture segmentation tasks.

## Supplementary Information

Supplementary Information.

## Data Availability

All data is available from author C.D. upon request.
