# Supplementary material for: Segmenting surface boundaries using luminance cues
Source: Sci Rep. 2021 May 12;11:10074. doi: 10.1038/s41598-021-89277-2 (PMC8115076; doi:10.1038/s41598-021-89277-2)

SUPPLEMENTARY MATERIAL

TITLE: "Segmenting surface boundaries using luminance cues"

AUTHORS: Christopher DiMattina [1,*] & Curtis L. Baker, Jr.[2]

[1]Computational Perception Laboratory & Department of Psychology
Florida Gulf Coast University, Fort Myers, FL, USA 33965-6565

[2]McGill Vision Research Unit, Department of Ophthalmology,
McGill University, Montreal, QC, Canada H3G1A4

TABLE S1

	BIC: Additive		BIC: Divisive		BIC_D - BIC_A	
	None	***Lapse***	***None***	***Lapse***	***None***	***Lapse***
CJD	-427.510	-430.820	-419.665	-423.020	7.845	7.800
ERM	-448.073	-451.383	-438.469	-441.779	9.605	9.605
KNB	-424.487	-427.797	-411.348	-414.658	13.139	13.139

Table S1: Bayes Information Criterion (BIC) for fits of Additive and Divisive SDT models to data from **Experiment 3**, with (*Lapse*) and without (*None*) lapse rates.

TABLE S2

Observer	neu	con	inc	χ^2	*p*
CJD	0.80	0.83	0.74	4.575	0.102
ERM	0.70	0.78	0.67	5.742	0.057
KNB	0.78	0.83	0.81	1.287	0.525
MCO	0.76	0.82	0.78	2.612	0.271
NRB	0.89	0.92	0.89	1.115	0.573
RCL	0.78	0.86	0.81	3.842	0.146
JCO	0.92	0.95	0.95	1.974	0.373
HAP	0.90	0.87	0.86	1.603	0.449
EMW	0.88	0.93	0.88	3.598	0.166
**POOLED**	**0.82**	**0.86**	**0.82**	**15.319**	**<0.001*** **

* $p < 0.05$, ** $p < 0.01$, *** $p < 0.001$

Table S2: Statistical tests (Pearson's χ^2**,** *df* = 2) of the hypothesis that the proportion correct (*Pc*) is identical for the *neutral* (**neu**), *congruent* (**con**), and *incongruent* (**inc**) conditions of **Experiment 4a**.

TABLE S3

**Observer**	**neu**	**con-0**	χ^2	p	**con-180**	χ^2	p
CJD	0.80	0.90	4.800	0.028*	0.75	0.982	0.322
ERM	0.70	0.83	5.905	0.015*	0.72	0.129	0.720
KNB	0.78	0.84	1.500	0.221	0.81	0.362	0.548
MCO	0.76	0.83	2.185	0.139	0.81	1.15	0.283
NRB	0.89	0.96	4.579	0.032*	0.87	0.142	0.706
RCL	0.78	0.86	2.736	0.098	0.85	2.068	0.150
JCO	0.92	0.97	3.241	0.072	0.92	0.022	0.883
HAP	0.90	0.88	0.280	0.597	0.86	1.061	0.303
EMW	0.88	0.98	8.422	0.004**	0.88	0.000	1.000
**POOLED**	**0.82**	**0.89**	**24.383**	**<0.001*** **	**0.83**	**0.288**	**0.592**

* $p < 0.05$, ** $p < 0.01$, *** $p < 0.001$

Table S3: Statistical comparisons (Pearson's χ^2, *df* = 1) of observer performance in the neutral (**neu**) case of **Experiment 4a** with each sub-condition of the congruent trials: Aligned-phase (**con-0**) and opposite-phase (**con-180**).

TABLE S4

**Observer**	**con-0**	**con-180**	χ^2	***p***
CJD	0.90	0.75	7.792	0.005**
ERM	0.83	0.72	3.470	0.063
KNB	0.84	0.81	0.312	0.577
MCO	0.83	0.81	0.136	0.713
NRB	0.96	0.87	5.207	0.022*
RCL	0.86	0.85	0.040	0.841
JCO	0.97	0.92	2.405	0.121
HAP	0.88	0.86	0.177	0.674
EMW	0.98	0.88	7.689	0.006**
**POOLED**	**0.89**	**0.83**	**15.732**	**<0.001*** **

* $p < 0.05$, ** $p < 0.01$, *** $p < 0.001$

Table S4: Statistical tests (Pearson's χ^2, $df = 1$) of the hypothesis that proportion correct is identical for phase-aligned (**con-0**) and opposite-phase (**con-180**) stimuli for the congruent case in **Experiment 4a**.

TABLE S5

Observer	isolated	masked
CJD	0.0041 [0.0035, 0.0055]	0.0100 [0.0083, 0.0165]
KNB	0.0043 [0.0039, 0.0048]	0.0083 [0.0072, 0.0098]
MXD	0.0056 [0.0048, 0.0066]	0.0113 [0.0094, 0.0170]

Table S5: Median LSB segmentation thresholds and non-parametric 95% confidence intervals (200 bootstraps) when the LSB is presented in isolation (**isolated**) and in the presence of an uninformative LTB masker (**masked**) having no boundary (equal number of B and W micro-patterns on each side). We see slightly higher LSB segmentation thresholds in the presence of the uninformative LTB masker, rising from about 0.5% to 1% Michelson Contrast.

TABLE S6

**Observer**	**neu**	**con**	**inc**	χ^2	***p***
CJD	0.87	0.83	0.73	13.189	0.001**
KNB	0.86	0.85	0.83	0.933	0.627
MXD	0.90	0.90	0.87	0.829	0.661
**POOLED**	**0.88**	**0.86**	**0.81**	**10.786**	**0.005****

* $p < 0.05$, ** $p < 0.01$, *** $p < 0.001$

Table S6: Statistical tests (Pearson's χ^2, $df = 2$) of the hypothesis that the proportion correct (*Pc*) is identical for the *neutral* (**neu**), *congruent* (**con**), and *incongruent* (**inc**) conditions of **Experiment 4b**.

TABLE S7

**Observer**	**neu**	**con-0**	χ^2	***p***	**con-180**	χ^2	***p***
CJD	0.87	0.95	4.605	0.032*	0.70	12.69	<0.001***
KNB	0.86	0.89	0.531	0.466	0.80	1.786	0.181
MXD	0.90	0.94	1.648	0.199	0.85	1.278	0.258
**POOLED**	**0.88**	**0.93**	**5.552**	**0.018***	**0.78**	**12.794**	**<0.001*** **

* $p < 0.05$, ** $p < 0.01$, *** $p < 0.001$

Table S7: Statistical comparisons (Pearson's χ^2, $df = 1$) of observer performance in the neutral (**neu**) case of **Experiment 4b** with each sub-condition of the congruent trials: aligned-phase (**con-0**) and opposite-phase (**con-180**).

TABLE S8

**Observer**	**con-0**	**con-180**	χ^2	***p***
CJD	0.95	0.70	21.645	<0.001***
KNB	0.89	0.80	3.092	0.079
MXD	0.94	0.85	4.310	0.038*
**POOLED**	**0.93**	**0.78**	**24.857**	**<0.001*** **

* $p < 0.05$, ** $p < 0.01$, *** $p < 0.001$

Table S8: Statistical tests (Pearson's χ^2, $df = 1$) of the hypothesis that proportion correct is identical for phase-aligned (**con-0**) and opposite-phase (**con-180**) stimuli for the congruent case in **Experiment 4b**.

TABLE S9

Observer	BIC_1	BIC_2	$BIC_2 – BIC_1$	IE ratio	DC response
CJD	-334.993	-316.758	18.235	0.00	26.7473
ERM	-375.381	-367.651	7.730	0.20	8.8932
KNB	-344.449	-309.400	35.049	0.25	4.4296
MCO	-353.603	-325.880	27.723	0.30	-0.0339
NRB	-277.090	-208.152	68.938	0.30	-0.0339
RCL	-312.702	-299.927	12.775	0.25	4.4296
JCO	-161.696	-156.019	5.6770	0.05	22.2837
HAP	-273.753	-236.640	37.113	0.20	8.8932
EMW	-267.175	-208.309	58.866	0.30	-0.0339

Table S9: Bayes Information Criteria (BIC) for model fits to data from **Experiment 4a**, for both one-stage (**BIC_1**) and two-stage (**BIC_2**) models. The two rightmost columns show the optimal values of the IE ratio for the first-stage filters in the two-stage model, as well as the DC response of this first-stage filter to uniform unit luminance.

TABLE S10

Observer	BIC_1	BIC_2	$BIC_2 – BIC_1$	IE ratio	DC response
CJD	-320.289	-287.982	32.307	0.05	22.2837
KNB	-318.614	-270.836	47.778	0.25	4.4296
MXD	-287.426	-222.493	64.933	0.20	8.8932

Table S10: Same as **Table S9** but for **Experiment 4b**.

SUPPLEMENTARY FIGURE CAPTIONS

Figure S1: Effects of density on LTB segmentation thresholds

Effects of micro-pattern density on segmentation thresholds for experienced observers (author CJD and naïve observers KNB, ERM). Plotted are means (circles) and 1 SEM (lines) obtained from N = 200 bootstrapped fits of the SDT psychometric function. We see that thresholds are slightly higher for 16 micro-patterns, and that similar performance is obtained for 32 and 64 micro-patterns.

Figure S2: Thresholds estimated with and without lapse rates

(**a**) Thresholds from **Experiment 1a**. We see nearly identical threshold estimates whether or not lapse rates are included in our PF definitions. (**b**) Thresholds from **Experiment 1b**.

Figure S3: Contrast thresholds for LSB segmentation

(**a**) Fits of SDT psychometric function to LSB segmentation performance for same observers shown in **Fig. 3a**. (**b**) Histogram of threshold for all observers.

Figure S4: Fits of one-stage model to LSB segmentation performance

Same as **Fig. 4b** in main text, but shows fits of the one-stage model to LSB segmentation data from **Experiment 1b**.

Figure S5: Fits of additive and divisive one-stage model

(**a**) Same as **Fig. 6b**, but with lapse rates estimated. (**b**) Divisive model fit to **Experiment 3** data accurately predicts performance in **Experiment 2**.

Figure. S6: Fits of models to Experiment 4a data

Same as **Fig. 8b** in main text, but for remaining observers.

Fig S7: Fits of the two-stage model (Fig. 8a) to Experiment 3 data

Fits of both additive and divisive instances of the two-stage model shown in **Fig. 8a** to psychophysical data obtained in **Experiment 3**. As with the one-stage model (**Fig. 6)**, we find much better fits with the divisive two-stage model.

Fig S8: Results from Experiment 4b

(**a**) Same as **Fig. 7a** in main text for **Experiment 4b**.

(**b**) Same as **Fig. 7b** in main text for **Experiment 4b**.

(**c**) Same as **Fig. 8b** in main text for **Experiment 4b**.

Supplementary Fig. S1

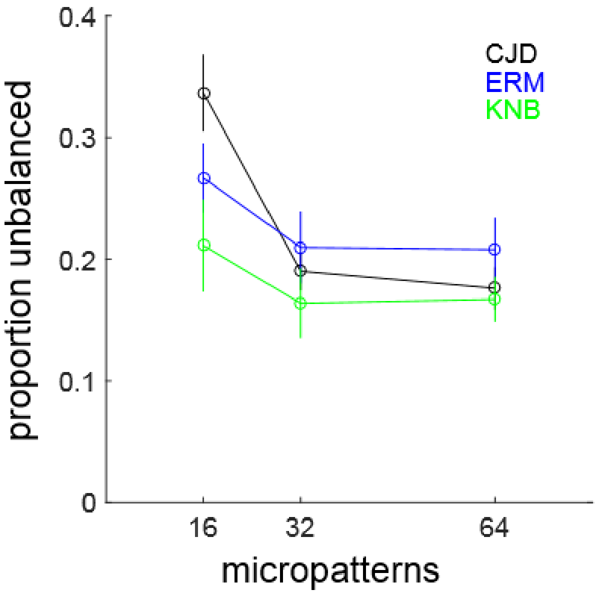

Supplementary Fig. S2

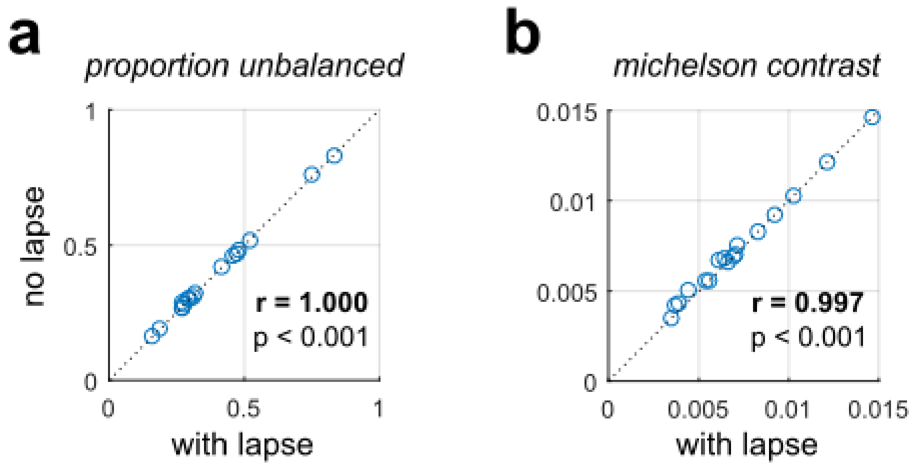

Supplementary Fig. S3

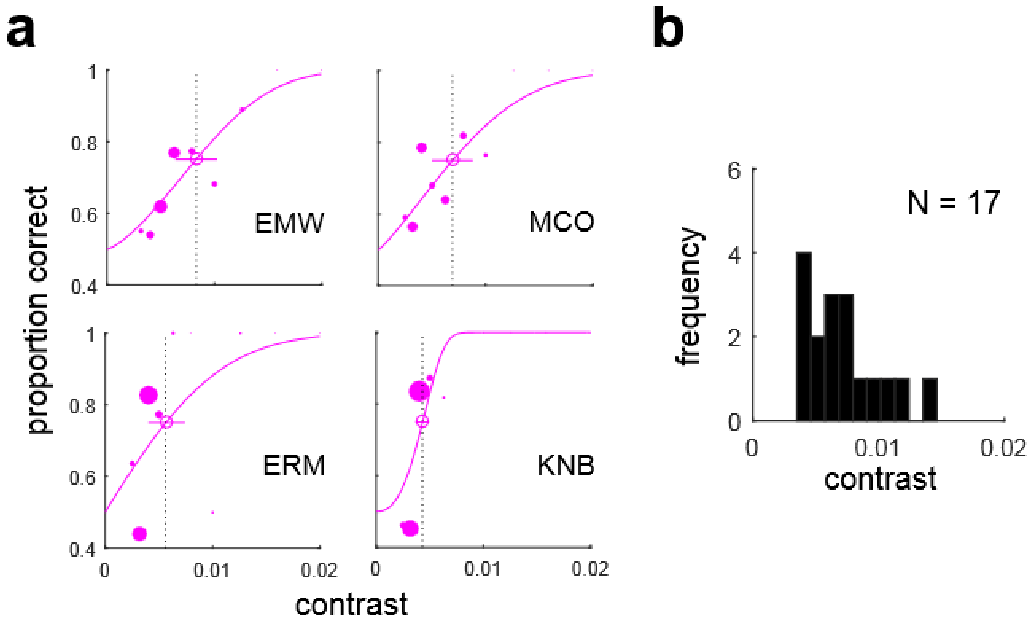

Supplementary Fig. S4

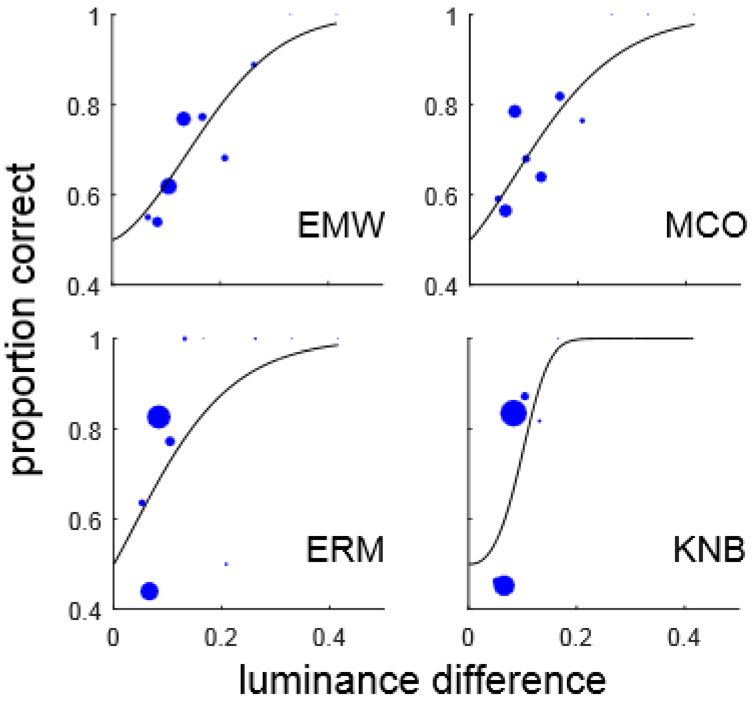

Supplementary Fig. S5

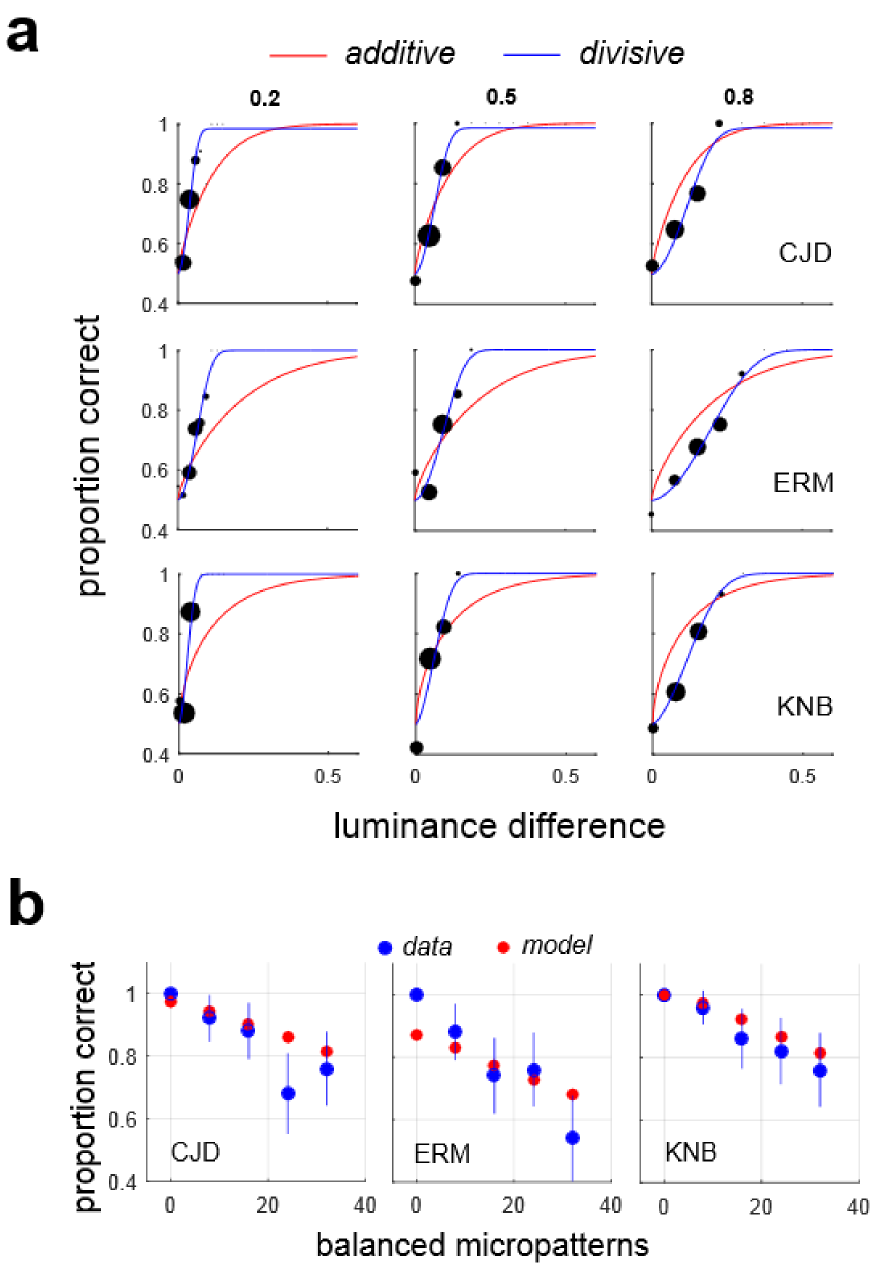

Supplementary Fig. S6

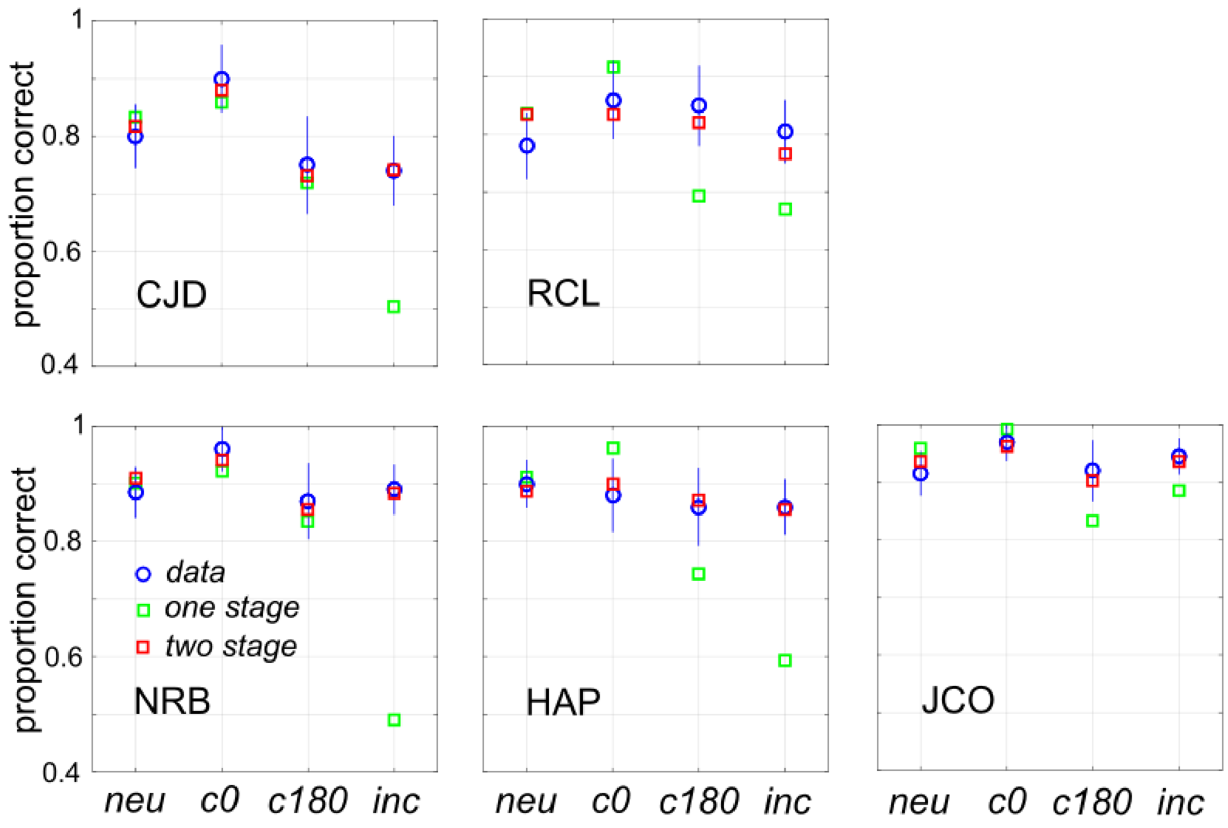

Supplementary Fig. S7

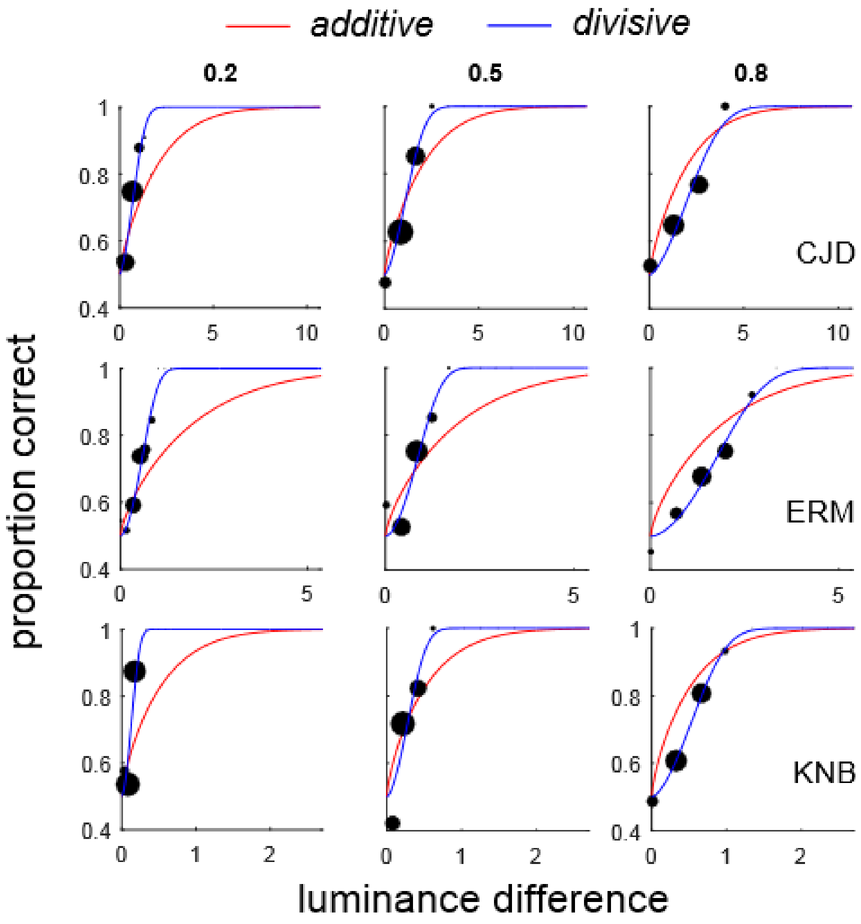

Supplementary Fig. S8

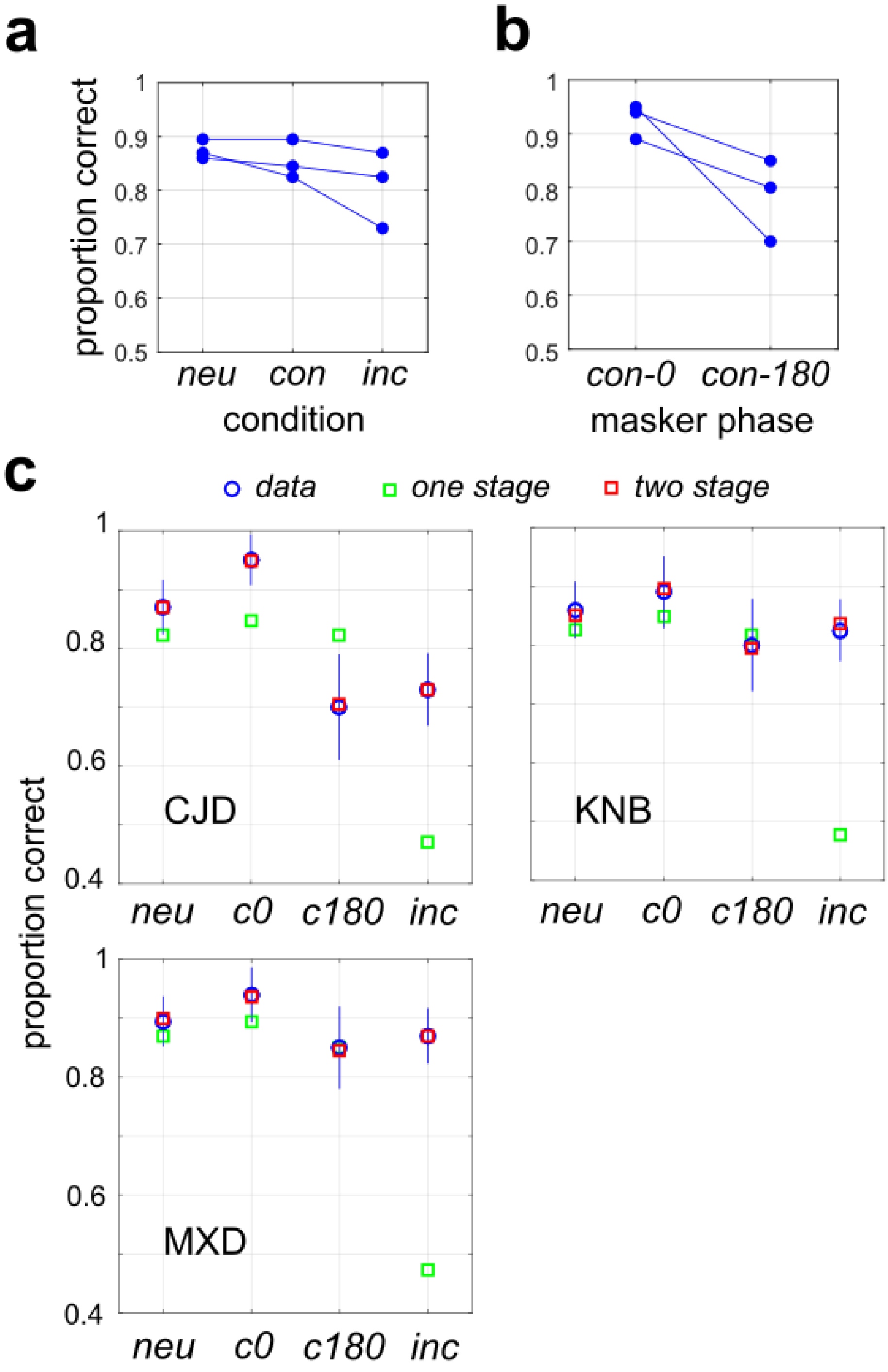

Supplement: Supplementary file 1 — Supplementary Information. [file 41598_2021_89277_MOESM1_ESM.pdf]
